# Designing Two Secure Keyed Hash Functions Based on Sponge Construction and the Chaotic Neural Network

**DOI:** 10.3390/e22091012

**Published:** 2020-09-10

**Authors:** Nabil Abdoun, Safwan El Assad, Thang Manh Hoang, Olivier Deforges, Rima Assaf, Mohamad Khalil

**Affiliations:** 1 Institut d’Electronique et des Télécommunications de Rennes (IETR), UMR CNRS 6164, Université de Nantes-Polytech, 44306 Nantes, France; nabil.abdoun@etu.univ-nantes.fr (N.A.); safwan.elassad@univ-nantes.fr (S.E.A.); 2 School of Electronics and Telecommunications, Hanoi University of Science and Technology, 1 Dai Co Viet, Hai Ba Trung, Hanoi 100000, Vietnam; 3 Institut d’Electronique et des Technologies du NuméRique, UMR CNRS 6164, INSA Rennes, 35700 Rennes, France; olivier.deforges@insa-rennes.fr; 4 LASTRE Laboratory, Lebanese University, Beirut, Lebanon; rima.assaf@ul.edu.lb (R.A.); mohamad.khalil@ul.edu.lb (M.K.)

**Keywords:** chaotic neural network, keyed hash functions, security analysis, speed analysis, sponge construction

## Abstract

In this paper, we propose, implement, and analyze the structures of two keyed hash functions using the Chaotic Neural Network (CNN). These structures are based on Sponge construction, and they produce two variants of hash value lengths, i.e., 256 and 512 bits. The first structure is composed of two-layered CNN, while the second one is formed by one-layered CNN and a combination of nonlinear functions. Indeed, the proposed structures employ two strong nonlinear systems, precisely a chaotic system and a neural network system. In addition, the proposed study is a new methodology of combining chaotic neural networks and Sponge construction that is proved secure against known attacks. The performance of the two proposed structures is analyzed in terms of security and speed. For the security measures, the number of hits of the two proposed structures doesn’t exceed 2 for 256-bit hash values and does not exceed 3 for 512-bit hash values. In terms of speed, the average number of cycles to hash one data byte (NCpB) is equal to 50.30 for Structure 1, and 21.21 and 24.56 for Structure 2 with 8 and 24 rounds, respectively. In addition, the performance of the two proposed structures is compared with that of the standard hash functions SHA-3, SHA-2, and with other classical chaos-based hash functions in the literature. The results of cryptanalytic analysis and the statistical tests highlight the robustness of the proposed keyed hash functions. It also shows the suitability of the proposed hash functions for the application such as Message Authentication, Data Integrity, Digital Signature, and Authenticated Encryption with Associated Data.

## 1. Introduction

Hash functions can be used in various applications such as Message Authentication, Digital Signature, Data Integrity, and Authenticated Encryption [1]. As a definition, a hash function *H* takes an input message *M*, and produces an output value *h*, named hash code, digital fingerprint, message digest, or simply hash. Precisely, the hash function *H* takes a bit sequence *M* (e.g., data, image, video, and file) with an arbitrary finite length, and produces a fixed length digest *h* of *u* bits. The digest acts as a kind of signature for the input data. Moreover, when the same hash function *H* is run for the same input message *M*, the same hash value *h* is obtained [2].

A cryptographic hash function employs an encryption algorithm in producing the output value *h*. The advantage of cryptographic hash functions is to meet some security requirements and to be immune against different attacks such as statistical, brute-force, and cryptanalytic attacks, etc. Recently, CNN based hash functions [3,4] attract the interest of research community because of the important properties of chaotic systems and neural networks related to the nonlinear security [5,6].

In general, chaos is a kind of deterministic random-like process generated by nonlinear dynamical systems. Chaos was given by Edward Lorenz [7], and its main properties have been investigated by a large community of research [8]. Chaotic systems are appropriate to be used in cryptographic hash algorithms due to their pertinent properties such as random-like behavior, sensitivity to tiny changes in initial conditions, and unstable periodic orbits. In addition, neural networks are powerful computational models, designed to simulate the human brain and adopted to solve many problems in different fields. Neural networks exhibit, by construction, many convenient properties to be used in cryptographic hash algorithms such as parallel implementation, flexibility, nonlinearity, one-way, data diffusion, and compression functions.

At first, some designers combine both these systems (chaos and neural network) in the *Merkle–D$\overset{˚}{a}$mgard* structure to build robust CNN hash functions [9,10]. In our previous work [2], Abdoun et al. designed, implemented, and analyzed the performance, in terms of security and speed, of two proposed keyed CNN hash functions based on the *Merkle–D$\overset{˚}{a}$mgard* (MD) construction with three output schemes, i.e., CNN–Matyas–Meyer–Oseas, Modified CNN–Matyas–Meyer–Oseas, and CNN–Miyaguchi–Preneel. However, the *Merkle–D$\overset{˚}{a}$mgard* construction has several vulnerabilities to some attacks such as Second preimage, Multicollisions, Herding, and Length extension attacks [11,12]. To resist these attacks, a new Secure Hash Algorithm called *SHA-3* [13] based on an instance of the *KECCAK* algorithm was selected as a winner of the National Institute of Standards and Technology (*NIST*) hash function competition in 2015 [13,14,15,16,17,18]. Indeed, the *SHA-3* family consists of four cryptographic hash functions such as, *SHA3-224*, *SHA3-256*, *SHA3-384*, and *SHA3-512* and two Extendable-Output Functions (XOFs) such as *SHAKE128* and *SHAKE256* [13]. For the XOFs, the length of the output can be chosen to meet the requirements of user applications. There are different structures being used to build various hash functions such as *Wide Pipe* [19], *Merkle–D$\overset{˚}{a}$mgard* [20,21], *Haifa* [22], *Fast Wide Pipe* [23], *Sponge* [24], etc. Indeed, a number of these existing structures are employed in the design of many popular hash functions. The *Merkle–D$\overset{˚}{a}$mgard* construction is used in the design of *MD5* [25] family like *SHA-1* [26], and *SHA-2* [27] standards, while the *Sponge* construction is used to design a new secured standard hash algorithm *SHA-3* [13], which will be used when the current standard *SHA-2* will be inevitably compromised. In our previous work [28], Abdoun et al. proposed, implemented, and analyzed the performance of a new structure for keyed hash function based on chaotic maps, neural network, and *Sponge* construction.

Since 2009, there are several lightweight cryptographic hash functions [29] proposed that are based on a *Sponge* construction such as *LightMAC* [30], *TuLP* [31], *SipHash* [32], *QUARK* [33], *PHOTON* [34], and *SPONGENT* [35].

In this paper, two robust keyed hash functions that contain a chaotic system (*CS*) and a CNN-based *Sponge* construction are proposed. In these two proposed structures, the input message *M* is hashed to a hash value *h* with a fixed length of bits equal to 256 or 512 bits. The combination of *Sponge* construction and *CNN* results the increase in the robustness of the proposed hash function. The proposed structures are based on the efficient CS [36]. The efficient CS in [36] produces pseudo-chaotic samples and those are used as the parameter values of the neural network. In addition, the proposed activation function of neural network is formed of two chaotic maps that are connected in parallel. The proposed CNN and CS ensure that our hash functions are more secure against different attacks in comparison with other hash functions that are based on *Sponge* construction. Indeed, the various experimental results and theoretical analysis demonstrate the effectiveness and prove that the proposed hash functions have very good statistical properties, high message sensitivity, high key sensitivity, strong collision resistance, and are immune against collision, preimage, and second preimage attacks [37].

The rest of the paper is organized as follows: Section 2 introduces a brief reminder of cryptographic hash function properties. Then, the general models of *Sponge* and *keyed-Sponge* constructions are presented. Section 3 describes in detail the proposed structures of the two keyed *CNN* hash functions based on *Sponge* construction with their important constitutive elements. Section 4 shows the results and analysis in terms of security and computational performance for the proposed hash functions, and comparison with the two standards *SHA-2* and *SHA-3*. Finally, in Section 5, conclusions for the contribution and the future work are given.

## 2. Preliminaries

### 2.1. Properties and Classification of Cryptographic Hash Functions

The cryptographic hash function *H* (noticed also as hash functions in the rest of paper) must verify the two implementation properties, i.e., *ease of computation* and *compression*, in addition to the three main security properties, i.e., preimage resistance (called one-way), second preimage resistance (called weak collision resistance), and collision resistance (called strong collision resistance).

### 2.2. Structures of Cryptographic Keyed Hash Functions Based on Sponge Construction

In this section, we describe the three phases of the *Sponge* construction, and then how to build *keyed-Sponge* hash functions from unkeyed *Sponge* construction.

#### 2.2.1. The Sponge Construction: Initialization, Absorbing and Squeezing Phases

**Figure 1:**
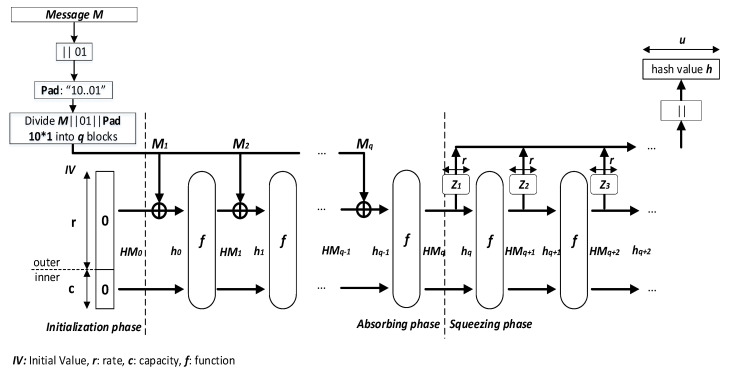
General architecture of the *Sponge* construction.

In Figure 1, the general structure of the unkeyed *Sponge* construction is shown and it has three phases: Initialization, Absorbing, and Squeezing. The unkeyed *Sponge* construction, which operates on a state $HM_{i}$(i≥0) of size *b* bits, builds a new hash function. These states are split into an outer part of *r*-bit size named *bitrate*, which is accessible externally, and an inner part *C* of *c*-bit size named *capacity*, which is hidden. The size called width *b*-bit is given by *b* = *r* + *c*. In the initialization phase, the initial value $IV=HM_{0}$ of *b*-bit size is set to 0. The input message *M* is padded and then split into *q* blocks of *r*-bit size. Next, in the absorbing phase, the *q* blocks of the entire message are absorbed on the basis of message block $M_{i}$ by message block $M_{i},\left(i=1,\cdots ,q\right)$. In the squeezing phase, the hash value *h* is obtained by squeezing out *r*-bit block by *r*-bit block.

Note that the security depends partially on the capacity *c*, while the speed of the construction relies partially on the bitrate *r*. In the absorption process, $HM_{i},\left(i=0,\cdots ,q-1\right)$, with *r*-bit size is xored with each message block $M_{i},\left(i=1,\cdots ,q\right)$, to become the input of the function *f*. If we increase the bitrate *r*, then more bits are absorbed at once and the process runs faster. However, the increase of the bitrate *r* implies the decrease in the capacity *c*, or the security is reduced. Thus, there is a trade-off between security and speed.

As mentioned before, the *KECCAK-p* family of permutations is the specialization of the *KECCAK-f* family:

(1) $$KECCAK-p\left[b,n_{r}\right]=KECCAK-f\left[b\right]$$

where $n_{r}$ is the number of rounds and *b* is the width. Therefore, the *KECCAK* family is denoted by *KECCAK*[*c*](*N*, *d*) as

(2) $$KECCAK\left[c\right]\left(N,d\right)=SPONGE[KECCAK-p\left[1600,24\right],1600-c,\;pad10^{*}1]\left(N,d\right),$$

where *N* is the concatenation of the initial message *M* with the suffix 01, or (*N* = *M* ‖ 01); *pad*$10^{*}1$ is the used padding rule explained below; *d* is the hash value length (*u* = *d*); and *Sponge*[.] is the sponge function. As we can see, for a given input message *M*, this equation is restricted to the case $n_{r}$ = 24 rounds and *b* = 1600 bits.

In particular, the four variants of *SHA-3* standard hash functions are defined from the *KECCAK*[*c*](*N*, *d*) function as follows:

$$\begin{array}{cc} \; & SHA3-256(M)=KECCAK[512](M\;\|\;01,256)\\ \; & SHA3-224(M)=KECCAK[448](M\;\|\;01,224)\\ \; & SHA3-512(M)=KECCAK[1024](M\;\|\;01,512)\\ \; & SHA3-384(M)=KECCAK[768](M\;\|\;01,384) \end{array}$$

In each case, the suffix 01 supports the domain separation; it distinguishes the *SHA-3* hash functions from the XOFs, where its suffix is 1111 (*N* = *M* ‖ 1111), and the capacity *c* is double the hash value length *u*, i.e., *c* = 2*u*.

Thus, to ensure that the obtained message (*M* ‖ 01) of arbitrary length is padded to become a bit string with the length of multiple of *r* bits, and a padding rule is necessary. Indeed, a simple padding rule with 0 is insufficient because the produced hash value will be vulnerable to various attacks due to the collision between all-zero latest message blocks.

#### 2.2.2. Unkeyed Sponge Construction to Keyed-Sponge Construction

**Figure 2:**
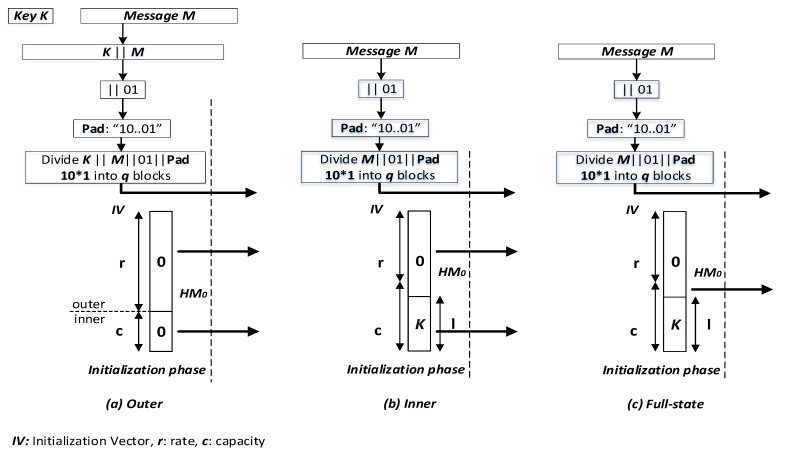
The three types of *keyed-Sponge* functions.

The unkeyed *Sponge* hash functions, which use an initial value *IV*, are transformed to *keyed-Sponge* hash functions, without any structural modification, by adding a secret key *K* as an additional entry to the structure. In the literature, three types of *keyed-Sponge* functions were reported as displayed in Figure 2:

1. The *Outer keyed-Sponge* (*OKS*) [38]: The input message is obtained by prepending the secret key *K* to the message *M*, i.e., K ‖ M as in Figure 2a.
2. The *Inner keyed-Sponge* (*IKS*) [39]: The inner part of the initial value *IV* contains the secret key *K* as in Figure 2b.
3. The *Full-State Keyed Sponge* (*FKS*) [40]: The inner part of the initial value *IV* contains the secret key *K* as *IKS*, but the input message *M* is absorbed over the entire *b*-bit state instead of absorbing it in the *r*-bit outer part only as in Figure 2c.

In the literature, the *OKS* and *IKS* hash functions were analyzed by *Andreeva* et al. [41], and *Naito* and *Yasuda* [42]. The *donkeySponge* construction employed the idea of the third type as in [43], and an analysis for only one output block was given by Gaži et al. [44]. A complete security analysis of the *FKS* was given by *Daemen* et al. [45] and *Mennink* et al. [40].

Under the security perspective, the same security level of *c* bits is achieved by the three modes, and there is no reason to take a key *K* of size |K| bits greater than the capacity *c* (|K|>c) [46]. However, in terms of the number of permutation evaluations, *OKS* and *IKS* are less efficient than *FKS*, that is, the absorption of *b*-bit input data at a time rather than *r* bits (r<b). Thus, we restrict our focus to *FKS* hash functions. There are several applications of the *keyed-Sponge* hash functions such as the *MAC* generation and the Bitstream encryption. For the first application, the *MAC* function is given by

(3) $$MAC_{K,IV}\left[M\right]:Z_{2}^{l}\times Z_{2}^{b}\times Z_{2}^{L}\to Z_{2}^{u},$$

where $Z_{2}$ is a binary sequence; *IV* is the initial value; *K* is the secret key; and |K|, *b*, *L*, and *u* are the lengths of the secret key *K*, the initial value *IV*, the message *M*, and the desired hash value *h*, respectively.

For the second application, the *STREAM* function is given by:

(4) $$STREAM_{K,IV}:Z_{2}^{l}\times Z_{2}^{b}\to Z_{2}^{\infty }.$$

In the next section, we introduce our proposed *keyed-Sponge CNN* hash functions.

## 3. Proposed Keyed-Sponge Chaotic Neural Network Hash Functions

**Figure 3:**
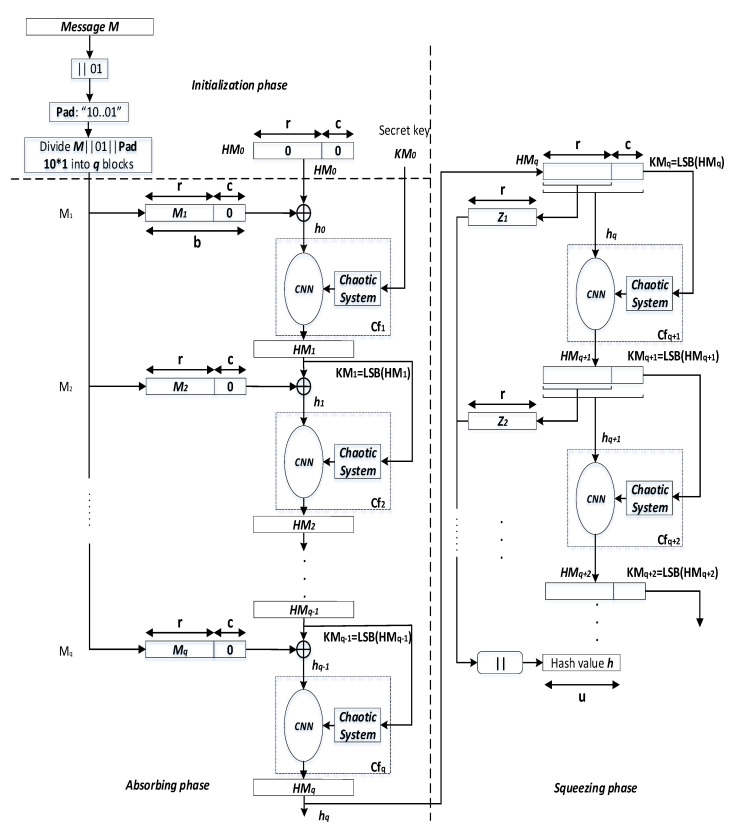
General architecture of the two proposed *keyed-Sponge CNN* hash functions.

The proposed *keyed-Sponge* hash functions are the chaotic functions $Cf_{i},\left(i\geq 1\right)$ that contain a *CS* and a *CNN* [47]. These chaotic functions use a padded message block $M_{i}\;\|\;0^{c},\left(i=1,\cdots ,q\right)$ of size *b*-bit, subkeys $KM_{i},\left(i\geq 1\right)$ of length 128 bits and a secret key $KM_{0}$ of length |K| = 160 bits and produce hash values with two variant lengths, 256 bits and 512 bits, depending on the value of *r* and *c* as shown in Figure 3.

The first *CNN* hash function is made up of a two-layered Neural Network called Structure 1, whereas the second hash function is made up of a one-layered Neural Network followed by a combination of Nonlinear (*NL*) functions called Structure 2 [28].

In the following subsection, the architecture of the two proposed *keyed-Sponge CNN* hash functions is described.

### 3.1. Description of the General Structure of the Two Proposed Keyed-Sponge CNN Hash Functions

The general structure of the proposed *keyed-Sponge CNN* hash functions (*KSCNN*[*c*](M ‖ 01, *u*)) is composed of three phases, i.e., Initialization, Absorbing, and Squeezing phases (see Figure 3).

#### 3.1.1. Phase 1: Initialization

This phase initializes the secret key $K=KM_{0}$ and the initial value $IV=HM_{0}$ to 0, and determines the values of *r* and *c* according to Table 1. In addition, the input message *M* is appended by the suffix 01, in this phase. Then, the appended message M||01 is padded using the function *Pad* (explained below), and divided into *q* blocks of the *r*-bit size, $M_{i}$ with (i=1,⋯,q).

For Structures 1 and 2, we adopt the same value of *c* like the standard *SHA-3*, i.e., *c* equal to 512 bits (like *SHA3-256*) for the 256-bit hash value, and *c* equal to 1024 bits (like *SHA3-512*) for the 512-bit hash value.

**Figure 4:**
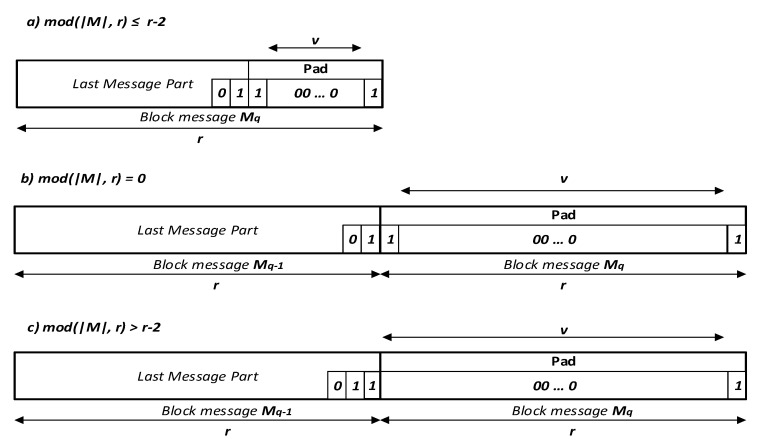
Padding rule in the two proposed *keyed-Sponge CNN* hash functions of the input message *M*.

We use the multi-rate padding *Pad* in our proposed hash functions, which appends a bit sequence 10∗1 of length *v* + 2 bits (a bit 1 followed by the minimum number *v* of bits 0, and lastly a bit 1), as shown in Equation (5):

(5) v=r−mod[((L+2)+2),r],

where *mod* is the modulo function and L=|M|. In general, we have three cases of padding as shown in Figure 4:

$$\begin{array}{cc} \; & \mathrm{Case}\;1:mod(|M+2|,r)\leq r-2;\\ \; & \mathrm{Case}\;2:mod(|M+2|,r)=0;\\ \; & \mathrm{Case}\;3:mod(|M+2|,r)>r-2. \end{array}$$

Now, let’s take a look at the three cases of padding, where *r* = 1088 bits as follows:

$$\begin{array}{cc} \mathrm{Case}\;1: & if\;L=3248\;bits:\\ \; & v=1088-mod[(3248+2)+2,1088]=12\;bits;\\ \mathrm{Case}\;2: & if\;L=3262\;bits:\\ \; & v=1088-mod[(3262+2)+2,1088]=1086\;bits;\\ \mathrm{Case}\;3: & if\;L=3261\;bits:\\ \; & v=1088-mod[(3261+2)+2,1088]=1087\;bits. \end{array}$$

Then, we divide the padded message into *q* blocks, and the obtained message is processed as a sequence of blocks:

(6) $$M_{1}\;\|\;M_{2}\;\|\;\ldots \;\|\;M_{q}=M\;\|\;01\;\|\;pad10^{*}1$$

#### 3.1.2. Phase 2: Absorbing

In the second phase, the *q* blocks of the message, $M_{i},\left(i=1,\cdots ,q\right)$, are absorbed, and each block is of *r* bits. Each message block $M_{i}$, (i=1,⋯,q), is padded by the sequence $0^{c}$. Then, the obtained blocks $M_{i}\;\|\;0^{c},\left(i=1,\cdots ,q\right)$ with the length of *b* bits are xored with the intermediate hash values $HM_{i-1}$, (i=1,⋯,q). It is noted that $HM_{0}$ is defined in the initialization phase ($HM_{0}=IV$). The obtained values from the xor operation $h_{i-1}$, (i=1,⋯,q), with the length of 1600 bits form the inputs of chaotic functions $Cf_{i},\left(i=1,\cdots ,q\right)$, in addition to the subkeys $KM_{i}$, (i=1,⋯,q−1), of 128 bits. For every *r*-bit input message block $M_{i}$, (i=1,⋯,q), the chaining variables $HM_{i}$, (i=1,⋯,q), of *b* bits (e.g., *b* = 1600 bits) are filled from the outputs of $Cf_{i},\left(i=1,\cdots ,q\right)$. $KM_{0}=K$ is the secret key of 160 bits for the first chaotic function $Cf_{1}$ [48]. For the other chaotic functions $Cf_{i},\left(i\geq 2\right)$, the subkeys $KM_{i},\left(i=1,\cdots ,q-1\right)$ are obtained from the Least Significant Bit (*LSB*) of $HM_{i},\left(i=1,\cdots ,q-1\right)$, or $KM_{i}=LSB\left(HM_{i}\right)$, (i=1,⋯,q−1). These subkeys $KM_{i}$(i=1,⋯,q−1) are used by the *CS* to generate initial conditions and the necessary parameters for the *CNN*. For the final chaotic function $Cf_{q}$, $HM_{q}$ forms the final hash value $h_{q}$ with the length equal to *b* bits as the output of the absorbing phase of the message *M*. The pseudo-code of the absorbing phase Algorithm 1 is presented below:
**Algorithm 1** The absorbing phase.**Require:**r<b$M_{1}\;\|\;M_{2}\;\|\;\ldots \;\|\;M_{q}\leftarrow Pad\left(M\;\|\;01\right)$$HM_{0}\leftarrow 0^{b}$**for** i = 1 to q **do**    $h_{i-1}\leftarrow HM_{i-1}⊕\left(M_{i}\;\|\;0^{c}\right))$
    $HM_{i}\leftarrow Cf_{i}\left(KM_{i-1},h_{i-1}\right)$
**end for****Return**${\left(\left\lfloor h_{q}\right\rfloor \right)}_{u}$.

#### 3.1.3. Phase 3: Squeezing

Squeezing phase is only used when the length of the hash value *u* is greater than the width *b*, i.e., u>b. In this case, the hash value $h_{q}$ of *b* bits generated by the absorbing phase is the input to the squeezing phase, and the obtained hash values $HM_{i}$, (i≥q), are sequentially forwarded to $Cf_{i},\left(i\geq q+1\right)$. For each $HM_{i},\left(i\geq q\right)$, we extract the *r* most significant bits to form $Z_{j},\left(j\geq 1\right)$, and the 128 least significant bits to produce the key $KM_{i},\left(i\geq q\right)$, for the *CS* of each $Cf_{i},\left(i\geq q+1\right)$. Finally, the *r*-bit size of all obtained values $Z_{j},\left(j\geq 1\right)$, are concatenated to constitute the final hash value *h* of the desired length of *u* bits as follows:

(7) $$h=Z_{1}\|Z_{2}\|Z_{3}||\ldots ={\left(\left\lfloor HM_{q}\right\rfloor \right)}_{r}\|{\left(\left\lfloor HM_{q+1}\right\rfloor \right)}_{r}\|{\left(\left\lfloor HM_{q+2}\right\rfloor \right)}_{r}\|\cdots$$

The obtained hash value *h* can be used as a Message Authentication Code (*MAC*) for Digital Signature (*DS*) and Authenticated Encryption (*AE*) applications [49,50]. The pseudo-code of the squeezing phase Algorithm 2 is given below:
**Algorithm 2** The squeezing phase.**Require:**u>b$Z_{1}\leftarrow {\left(\left\lfloor HM_{q}\right\rfloor \right)}_{r}$$h\leftarrow Z_{1}$j←2**for** i = q+1, … **do**    **while** |h|<u **do**        $h_{i-1}\leftarrow HM_{i-1}$
        $HM_{i}\leftarrow Cf_{i}\left(KM_{i-1},h_{i-1}\right)$
        $Z_{j}\leftarrow {\left(\left\lfloor HM_{i}\right\rfloor \right)}_{r}$
        $h\leftarrow h\;\|\;Z_{j}$
        j←j+1
    **end while****end for****Return**${\left(\left\lfloor h\right\rfloor \right)}_{u}$.

In the next paragraph, the proposed *CS* will be used in the chaotic functions $Cf_{i},\left(i\geq 1\right)$, to generate the necessary parameters and initial conditions for *CNN* as described above.

### 3.2. Detailed Description of the Proposed Chaotic System

**Figure 5:**
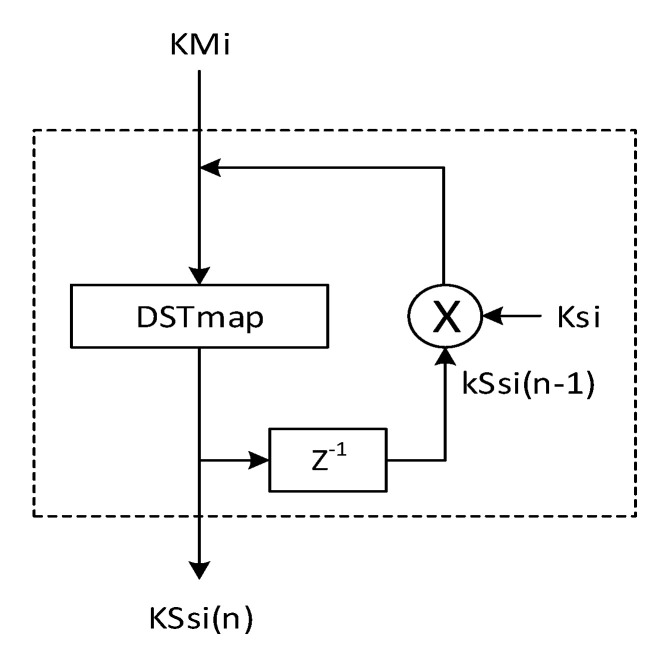
Structure of the ith *Chaotic System* used in the two proposed structure of *keyed-Sponge CNN* hash functions.

As shown in Figure 5, the proposed *CS* is a simple version of that given by *S. El Assad* and *H. Noura* [36]. It is based on the Discrete Skew Tent map (*DSTmap*) in Equation (8) as

(8) $$\begin{array}{cc} \; & KSs\left(n\right)=DSTmap\left(KSs\left(n-1\right),\;Q1\right)=\left\{\begin{array}{cc} 2^{N}\times \frac{KSs(n-1)}{Q1} & if\;0<KSs(n-1)<Q1\;\\ 2^{N}-1 & if\;KSs(n-1)=Q1\;\\ 2^{N}\times \frac{2^{N}-KSs\left(n-1\right)}{2^{N}-Q1} & if\;Q1<KSs\left(n-1\right)<2^{N} \end{array}\right. \end{array}$$

where *N* is the finite precision equal to 32 bits; and *Q1* is the control parameter of *DSTmap*. *KSs*(*n* − 1) and *KSs(n)* are the outputs of *DSTmap* at the ${\left(n-1\right)}^{th}$ and $n^{th}$ iterations, respectively. The value range of *Q1*, *KSs*(*n* − 1), and  *KSs(n)* is from 1 to $2^{N}-1$. The secret key *K* of the first input block message, $M_{1}$, is represented by the following equation:

(9) K={KSs1(−1), Ks1, KSs1(0), Q1, Us},

where KSs1(−1), Ks1, KSs1(0), Q1, and Us are parts of the secret key *K*. Us is only used for generation of the first sample. The components of the secret key *K* are samples of 32 bits, and its size is:

(10) |K|=|KSs1(−1)|+|Ks1|+|KSs1(0)|+|Q1|+|Us|=160(bits)

### 3.3. Keyed-Sponge Hash Functions Based on Two-Layered CNN Structure (Structure 1)

**Figure 6:**
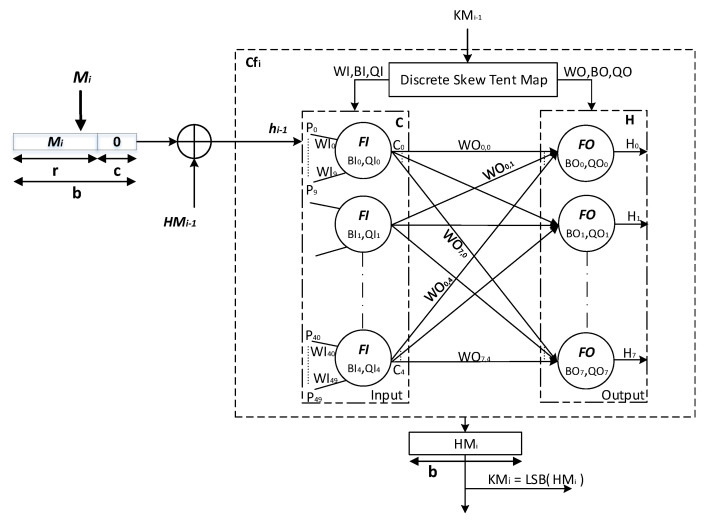
Detailed architecture of the ith chaotic function in the proposed two-layered *KSCNN* hash function.

The structure of the chaotic function $Cf_{i}$ for *KSCNN*[512] and *KSCNN*[1024] is shown in Figure 6. It contains two layers of neurons, i.e., a *CNN* input layer of five neurons and a *CNN* output layer of eight neurons. The necessary samples, Key Stream *KS*, are generated by the *CS* to supply the both layers. The *KS* is composed as follows:

(11) KS={BI,WI,QI,BO,WO,QO}

The size of KS must be:

(12) |KS|=|BI|+|WI|+|QI|+|BO|+|WO|+|QO|=129samples,

where |BI| = 5 samples, *|WI| = 50 samples, |QI| = 10 samples, |BO| = 8 samples*, |WO| = 40 *samples* and *|QO| = 16 samples*. Each component has 32 bits in length.

**Figure 7:**
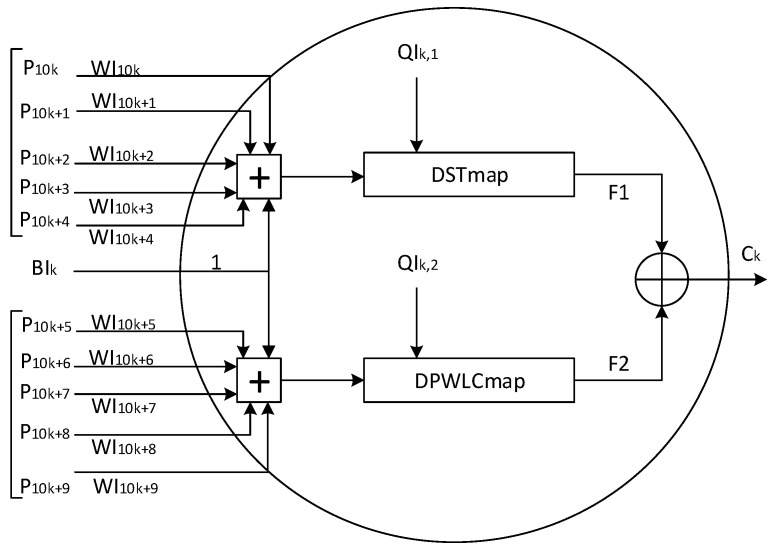
Detailed architecture of the kth neuron at the input layer of the two proposed *KSCNN* hash functions.

Indeed, all neurons of the two *CNN* layers use the same activation function with different number of inputs. For the input layer, each neuron has 10 inputs receiving data from $h_{i},\left(i=0,\cdots ,q-1\right)$ as displayed in Figure 6 and Figure 7. In addition, for (k=0,⋯,4), the first five inputs $P_{j}$, (j=10k,⋯,10k+4), of each neuron are weighted by the $WI_{j}$, (j=10k,⋯,10k+4), and then added together with the bias $BI_{k}$ (weighted by 1), to form the input of the chaotic map *DSTmap*. The last five inputs $P_{j}$, are weighted by $WI_{j},\left(j=10k+5,\cdots ,10k+9\right)$, and then combined together with the same bias $BI_{k}$ to form the input of the chaotic map *DPWLCmap*. All inputs $P_{j}$, biases $BI_{k}$ and weights $WI_{j}$ are samples (integer values) of 32 bits. $QI_{k,1}$ and $QI_{k,2}$ are the control parameters of *DSTmap* and *DPWLCmap*, respectively. The biases $BI_{k}$, (k=0,⋯,4), are necessary in case the input message is null as seen in Figure 7. The chaotic map *DPWLCmap* is realized as follows:

(13) $$KSp\left(n\right)=DPWLCmap\left(KSp\left(n-1\right),\;Q2\right)=\left\{\begin{array}{cc} 2^{N}\times \frac{KSp(n-1)}{Q2} & if\;0<KSp(n-1)\leq Q2;\;\\ 2^{N}\times \frac{KSp(n-1)-Q2}{2^{N-1}-Q2} & if\;Q2<KSp\left(n-1\right)\leq 2^{N-1};\;\\ 2^{N}\times \frac{2^{N}-KSp\left(n-1\right)-Q2}{2^{N-1}-Q2} & if\;2^{N-1}<KSp\left(n-1\right)\leq 2^{N}-Q2;\;\\ 2^{N}\times \frac{2^{N}-KSp\left(n-1\right)}{Q2} & if\;2^{N}-Q2<KSp\left(n-1\right)\leq 2^{N}-1;\;\\ 2^{N}-1-Q2 & otherwise; \end{array}\right.$$

where *KSp*(*n* − 1) and *KSp(n)* are the outputs of *DPWLCmap* at the ${\left(n-1\right)}^{th}$ and $n^{th}$ iterations, respectively; *N* is the number of bits defining the finite precision, N=32 bits; *Q2* is the control parameter; *KSp*(*n* − 1), *KSp(n)* and *Q2* range between 1 to $2^{N-1}$.

After computation, the two outputs of *DSTmap* and *DPWLCmap* are xored together to produce the output of neurons represented by $C_{k}$, (k=0,⋯,4), which is presented by the following equation:

(14) $$\begin{array}{c} C_{k}=mod\left\{\left[F1+F2\right],\;2^{N}\right\}\;where\left\{\begin{array}{c} F1=DSTmap\{mod\left(\left[\sum_{j=10k}^{10k+4}\left(WI_{j}\times P_{j}\right)\right]+\;BI_{k},2^{N}\right),\;QI_{k,1}\},\\ F2=DPWLCmap\{mod\left(\left[\sum_{j=10k+5}^{10k+9}\left(WI_{j}\times P_{j}\right)\right]+BI_{k},2^{N}\right),\;QI_{k,2}\}. \end{array}\right. \end{array}$$

**Figure 8:**
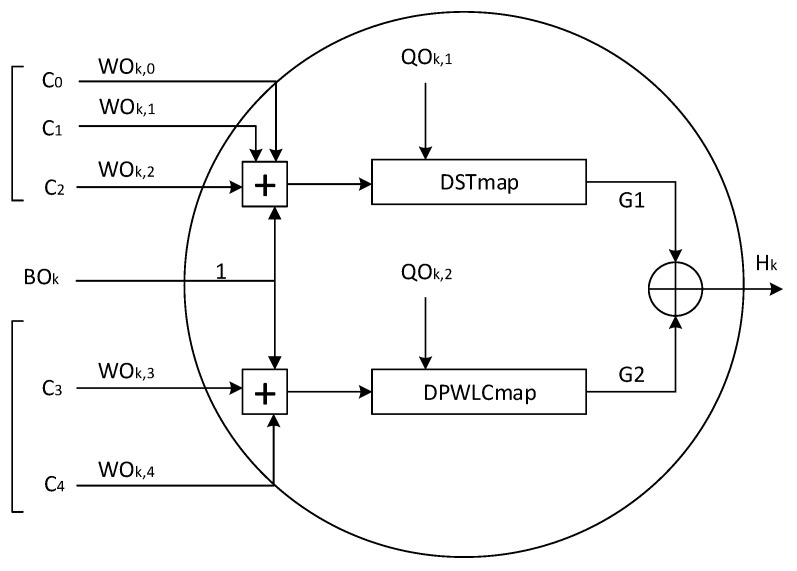
Detailed architecture of the kth neuron at the output layer of the proposed two-layered *KSCNN* hash functions.

At the output layer, each neuron has five inputs, $WO_{k,j}\times C_{j},\left(k=0,\cdots ,7;j=0,\cdots ,4\right)$, where *k* represents the index of output neurons, *j* represents the index of input neurons; $WO_{k,j}$, (k=0,⋯,7;j=0,⋯,4), are the weights associated with the connections between output and input layers, and $C_{j}$, (j=0,⋯,4) are the outputs of neurons at the input layer; $WO_{k,j}$, (k=0,⋯,7;j=0,⋯,4), and $C_{j}$, (j=0,⋯,4), both are samples of 32-bit length. As presented in Figure 8 for the inputs of each neuron at the output layer, the outputs of the first three neurons at the input layer, $C_{0}$, $C_{1}$ and $C_{2}$, are fed to the chaotic map *DSTmap*, and the last two outputs $C_{3}$ and $C_{4}$ from the input layer are sent to the chaotic map *DPWLCmap*. After computation, the outputs of chaotic maps *DSTmap* and *DPWLCmap* are xored together to generate the output of the neuron, given by the following equation:

(15) $$\begin{array}{c} H_{k}=mod\left\{\left[G1+G2,\;2^{N}\right]\right\}\;where\left\{\begin{array}{c} G1=DSTmap\{mod\left(\left[\sum_{j=0}^{2}\left(WO_{k,j}\times C_{j}\right)\right]+BO_{k},2^{N}\right),\;QO_{k,1}\},\\ G2=DPWLCmap\{mod\left(\left[\sum_{j=3}^{4}\left(WO_{k,j}\times C_{j}\right)\right]\;+\;BO_{k},2^{N}\right),\;QO_{k,2}.\} \end{array}\right. \end{array}$$

Here, the control parameters $QO_{k,1}$, $QO_{k,2}$, (k=0,⋯,7), and the biases $BO_{k}$, (k=0,⋯,7), used by the two chaotic maps, are also samples of 32 bits in length.

Finally, the output layer of the proposed structure is iterated seven times to produce the intermediate hash values with the length *b* = ⌊7×8×32⌋ bits.

### 3.4. Keyed-Sponge Hash Functions Based on One-Layered CNN and One NL Output Layer (Structure 2)

**Figure 9:**
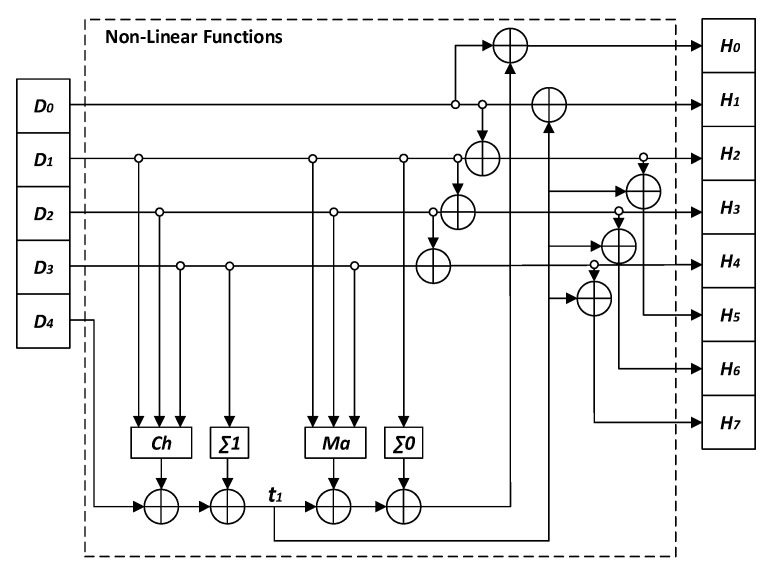
Detailed structure of *NL* Functions block.

The architecture of the second proposed *KSCNN* hash function uses the same input *CNN* layer as that in Structure 1, and the second layer is replaced by *NL* functions. The *NL* functions are similarly used in *SHA-2* as displayed in Figure 9. The *CS* generates the necessary samples to supply the *CNN* of each $Cf_{i}$, (i≥1) as

(16) KS={BI,WI,QI,WO},

and its size is

(17) $$\begin{array}{cc} \left|KS\right| & =|BI|+|WI|+|QI|+|WO|=70\;samples. \end{array}$$

Here, |WO| = 5 samples instead of 40 samples as used in Structure 1.

**Figure 10:**
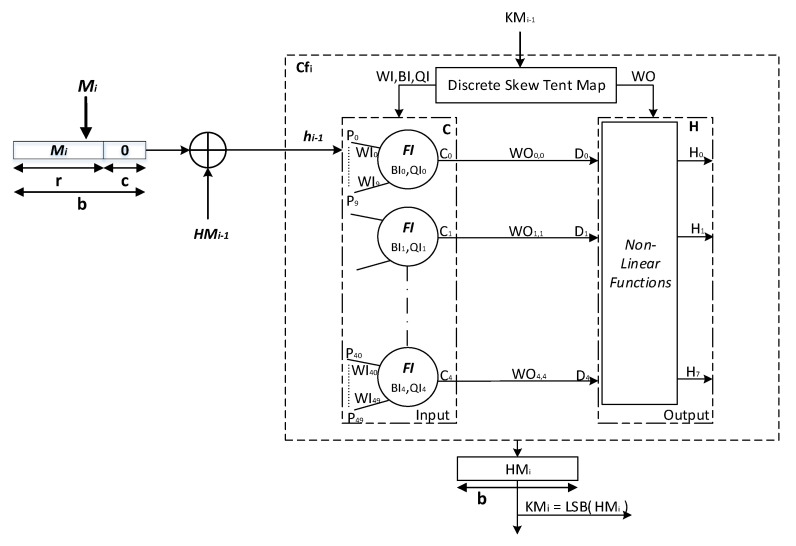
Detailed architecture of the ith chaotic function in the proposed *keyed-Sponge* hash function based on one-layered *NL CNN*.

The outputs of neurons at the input layer $C_{k}$, (k=0,⋯,4) are calculated by Equation (14). As seen in Figure 10, the outputs of neurons are weighted by $WO_{k,k}$, (k=0,⋯,4), to form the inputs $D_{k},\left(k\;=\;0,\cdots ,4\right)$ for the *NL* functions of the output layer; $D_{k}=WO_{k,k}\times C_{k},\left(k=0,\cdots ,4\right)$. The outputs $H_{k},\left(k=0,\cdots ,7\right)$ are calculated by the following equations:

(18) $$\left\{\begin{array}{c} H_{0}=D_{0}⊕t_{1}⊕Maj\left(D_{1},D_{2},D_{3}\right)⊕\Sigma 0\left(D_{1}\right),\\ H_{1}=t_{1}⊕D_{0},\\ H_{2}=D_{0}⊕D_{1},H_{3}=D_{1}⊕D_{2},H_{4}=D_{2}⊕D_{3},\\ H_{5}=D_{0}⊕D_{1}⊕t_{1},\\ H_{6}=D_{1}⊕D_{2}⊕t_{1},\\ H_{7}=D_{2}⊕D_{3}⊕t_{1},\\ where\;t_{1}=Ch\left(D_{1},D_{2},D_{3}\right)⊕D_{4}⊕\Sigma 1\left(D_{3}\right), \end{array}\right.$$

where $H_{k}$, (k=0,⋯,7) are values of 32-bit length and $D_{k}$, (k=0,⋯,4) are truncated to 32 bits. The four *NL* functions, *Maj*, *Ch*, Σ0 and Σ1, are defined by the equations as

(19) $$\left\{\begin{array}{c} Maj\left(D_{1},D_{2},D_{3}\right)=\left(D_{1}\wedge D_{2}\right)⊕\left(D_{1}\wedge D_{3}\right)⊕\left(D_{2}\wedge D_{3}\right),\\ Ch\left(D_{1},D_{2},D_{3}\right)=\left(D_{1}\wedge D_{2}\right)⊕\left(\neg D_{1}\wedge D_{3}\right),\\ \Sigma 0\left(D_{1}\right)=ROTR^{2}\left(D_{1}\right)⊕ROTR^{13}\left(D_{1}\right)⊕ROTR^{22}\left(D_{1}\right),\\ \Sigma 1\left(D_{3}\right)=ROTR^{6}\left(D_{3}\right)⊕ROTR^{11}\left(D_{3}\right)⊕ROTR^{25}\left(D_{3}\right),\\ ROTR^{n}\left(x\right)=\left(x\gg n\right)\vee \left(x\ll \left(32-n\right)\right), \end{array}\right.$$

where the denotations are ¬: NOT logic, ∧: AND logic, ∨: OR logic, ⊕: XOR logic, ≪: binary shift left operation, and ≫: binary shift right operation.

To compute the intermediate hash values, the output layer is iterated $n_{r}$ times firstly, while the value of $n_{r}$ (1, 2, 4, 8, 16, and 24) depends on the desired security level. The obtained results given in the performance section indicate that $n_{r}$ = 8 rounds is sufficient. Then, with fixed $n_{r}$, we again iterate the output layer seven times to obtain the desired length of the intermediate hash values as done in Structure 1.

## 4. Performance Analysis

In order to evaluate the performance of *KSCNN*[512] and *KSCNN*[1024], the performance analysis focuses on the security and the number of needed cycles per byte (*NCpB*). In addition, we compare the obtained performance with the standard hash algorithm *SHA-3*. First, we analyze the preimage resistance (one-way property) of the proposed structures. Then, we evaluate the statistical tests such as the collision resistance, the distribution of hash value, the sensitivity of hash value *h* to the message *M* and the sensitivity of hash value *h* to the secret key *K*, and the diffusion effect. In addition, we study the immunity of the proposed structures against the brute-force and cryptanalytic attacks. The detailed description of these tests is presented in our previous work [47]. For that, we just resume in this section the necessary test description to interpret the obtained results.

### 4.1. One-Way Property

According to Equations (14) and (15), for a hash value *h*, it is highly difficult to retrieve the secret key *K* and the message *M*. For a given secret key *K*, the attacker tries to find the message *M* using the brute force attack (as explained in the Section 4.3.1), such that its hash is equal to a given hash value. On average, an attacker tries $2^{u-1}$ values of the message, to find the hash value *h* of length *u* (*u* is equal to 256 or 512 bits). Nowadays, with such lengths, this attack is infeasible [51,52].

### 4.2. Statistical Tests

In this sub-section, we implement and analyze the different statistical tests.

#### 4.2.1. Collision Resistance Analysis

This statistical test quantitatively evaluates the collision resistance [51]. For that, given a hash value *h* of a random message *M* in the ASCII format $h=\{c_{1},c_{2},\cdots ,c_{s}\}$, and its corresponding $h^{\prime }\;=\;\left\{c_{1}^{\prime },c_{2}^{\prime },\cdots ,c_{s}^{\prime }\right\}$ obtained with one bit flipping of the same message *M*, we calculate the number of hits ω as follows:

(20) $$\omega =\sum_{i=1}^{s}f\left(T\left(c_{i}\right),T\left(c_{i}^{{}^{\prime }}\right)\right),$$

where the function

(21) $$f\left(x,y\right)=\left\{\begin{array}{cc} 0 & \;\;\;\mathrm{if}\;x\neq y;\\ 1 & \;\;\;\mathrm{if}\;x=y. \end{array}\right.$$

The value $s=\frac{u}{8}$, and T(.) is the function that converts the entries to their equivalent decimal values.

In theory, the relation between a number of tests and a number of hits ω=0,1,2,⋯,s as mentioned in [53] that

(22) $$W_{J}\left(\omega \right)=J\times Prob\left\{\omega \right\}=J\frac{s!}{\omega !(s-\omega )!}{\left(\frac{1}{2^{k}}\right)}^{\omega }{\left(1-\frac{1}{2^{k}}\right)}^{s-\omega },$$

where *J* represents the number of independent experiments. These theoretical values of $W_{J}\left(\omega \right)$ according to Equation (22) are given in Table 2 and Table 3 for hash values with the lengths of 256 and 512 bits, respectively.

For the two lengths of hash values, the obtained results in Table 4 indicate that the number of rounds $n_{r}$ = 8 and $n_{r}$ = 24 give the best results. Indeed, for 256-bit hash value length with $n_{r}$ = 8, there are two hits for 17 tests, one hit for 244 tests, and zero hits for 1787 tests. For $n_{r}$ = 24, there are two hits for 11 tests, one hit for 213 tests, and zero hits for 1824 tests. Similar behavior is obtained for the 512-bit hash value with a slight increase in the number of hits.

In Table 5, we summarize the obtained number of hits ω=0, 1, 2, 3, 4 for the two proposed structures. As expected, we obtain comparable results. The absolute difference *d* of two hash values is calculated as

(23) $$d=\sum_{i=1}^{s}\left|T\left(c_{i}\right)-T\left(c_{i}^{{}^{\prime }}\right)\right|.$$

The mean, mean/character, minimum, and maximum of *d* are presented in Table 6. It is clear that the values of mean/character are close to the expected ones as observed from the obtained results, evaluated by Equation (24) that are equal to 85.33 for 256-bit hash value length (*L* = 256) and equal to 170.66 for 512-bit hash value length [54]:

(24) $$E\left[T\left(c_{i}\right)-T\left(c_{i}^{{}^{\prime }}\right)\right]=\frac{1}{3}\times L$$

#### 4.2.2. Hash Value Distribution

Theoretically, the hash value *h*, produced by a hash function *H*, should be uniformly distributed in the entire output range. For this purpose, we execute the following test for a given message *M* as:

“With the wide application of Internet and computer technique, information security becomes more and more important. As we know, hash function is one of the cores of cryptography and plays an important role in information security. Hash function takes a message as input and produces an output referred to as a hash value. A hash value serves as a compact representative image (sometimes called digital fingerprint) of input string and can be used for data integrity in conjunction with digital signature schemes.”

**Figure 11:**
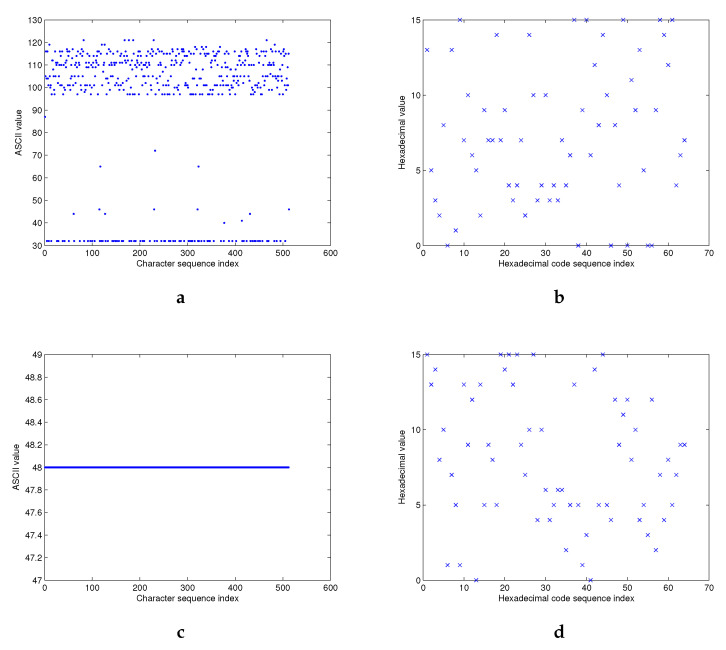
Hash value distribution for Structure 1 with |h| = 256 bits. (**a**) ASCII values of message *M*, (**b**) Hexadecimal hash value *h* of *M*, (**c**) Zero message, and (**d**) Hexadecimal hash value *h* of the zero message.

The hash value *h* is computed using Structures 1 and 2 with the 256-bit and 512-bit hash value lengths. In Figure 11, we exhibit the ASCII values of the message *M* (Figure 11a), and its hexadecimal hash value *h* (Figure 11b) according to their index of positions.

As predicted, the distribution of the original message is located around a small area, while the distribution of hexadecimal hash value looks like a mess. The distribution of the hash value *h* (Figure 11d) is also verified, even under the worst case of zero input message (Figure 11c). Similar results are obtained for the two proposed structures with their two variant hash output lengths.

#### 4.2.3. Sensitivity of Hash Value *h* to the Input Message M

A hash function *H* is very sensitive to an input message *M*. It means that a small change in its input will generate a totally different hash value $h_{i}$. To this end, for a given secret key *K*, the hash value $h_{i}$ in hexadecimal, the number of changed bits $B_{i}\left(h,h_{i}\right)$, and the sensitivity of the hash value *h* to the original message *M* are measured by Hamming Distance $HD_{i}\left(h,h_{i}\right)\left(\%\right)$ for the two proposed structures with their two variants of hash value lengths of 256 and 512 bits as

(25) $$B_{i}\left(h,h_{i}\right)=\sum_{k=1}^{\left|h\right|}\left[h\left(k\right)⊕h_{i}\left(k\right)\right]\;bits,$$

and

(26) $$HD_{i}{\left(h,h_{i}\right)}_{\%}=\frac{B_{i}\left(h,h_{i}\right)}{\left|h\right|}\times 100\%.$$

The different message variants are obtained under the following six conditions:

*Condition 1*: The input message *M* is the one given in Section 4.2.2.
*Condition 2*: The first character W in the input message is changed to X.
*Condition 3*: The word With in the input message is changed to Without.
*Condition 4*: The dot at the end of the input message is changed to the comma.
*Condition 5*: A blank space at the end of the input message is added.
*Condition 6*: We exchange the first block $M_{1}$

“With the wide application of Internet and computer technique, information security becomes more and more important. As we know, hash function is one of the cores of cryptography and plays an important role in information security. Hash function takes a mes,”

with the second block $M_{2}$

“sage as input and produces an output referred to as a hash value. A hash value serves as a compact representative image (sometimes called digital fingerprint) of input string and can be used for data integrity in conjunction with digital signature schemes.”

With each condition, Table 7 shows the obtained results of $h_{i},B_{i}$, and $HD_{i}\left(\%\right)$ for the 256-bit hash value. Similar results are obtained for |h| = 512 bits. In Table 8, the obtained results for the two structures with their two lengths of 256 and 512 bits are compared. All the results are close to the expected values ($B_{i}$ = 128 bits for the 256-bit hash value length, $B_{i}$ = 256 for the 512-bit hash value length, and $HD_{i}$ = 50% for all proposed structures), demonstrating the high sensitivity to the input message *M* for the two proposed structures.

#### 4.2.4. Sensitivity of Hash Value *h* to the Secret Key *K*

A hash function *H* is highly sensitive to the secret key *K* when a slight change in *K* produces a completely different hash value $h_{i}$. Here, for the previous message *M* with each of the five following conditions and for the two proposed structures with their two variants of hash value length 256 and 512 bits, we calculate the hash value $h_{i}$ (hexadecimal), the number of changed bits $B_{i}\left(h,h_{i}\right)$ (bits), and the sensitivity of the hash value *h* to the secret key *K* measured by Hamming Distance $HD_{i}\left(h,h_{i}\right)\left(\%\right)$:

*Condition 1*: The original secret key *K* is used.
In each of these conditions, we flip the *LSB* in the aforementioned parameters and initial conditions.
*Condition 2*: The initial condition *KSs(0)* in the secret key is changed.
*Condition 3*: The parameter *Ks* in the secret key is changed.
*Condition 4*: The initial condition *KSs(−1)* in the secret key is changed.
*Condition 5*: The control parameter *Q1* in the secret key is changed.

Table 9 presents the obtained results of $h_{i},B_{i}$, and $HD_{i}\left(\%\right)$ for 256-bit hash value length. Comparable results are obtained for |h| = 512 bits. We compare the results of the two proposed structures for two lengths of 256 and 512 bits in Table 10. All results obtained are close to the expected values ($B_{i}$ = 128 bits for the 256-bit hash value length, $B_{i}$ = 256 for the 512-bit hash value length, and $HD_{i}$ = 50% for all proposed structures), demonstrating the high sensitivity to the secret key *K* of the two proposed structures.

#### 4.2.5. Statistical Analysis of the Diffusion Effect

We obtain the optimal value of diffusion effect when flipping any bit in the input message *M* that causes a change of each output bit (binary format) in the hash value *h* with a probability of 50% [55]. This is often mentioned as the *Strict Avalanche Criterion* (*SAC*) in literature [56].

To quantify the performance of Structures 1 and 2 with their variants of hash output lengths of 256 and 512 bits, we execute the following diffusion test.

First, the hash value *h* for the previous message *M* is generated. Next, a new hash value *h’* for the same message *M* with one randomly changed bit is produced. Then, the number of bits changed $B_{i}$ between the two obtained hash values *h* and *h’* is calculated. This experiment is repeated *J* times, with *J* = 512, 1024, and 2048. Finally, we compute the six following statistical tests as below:

1. Minimum number of bits changed:
  $B_{min}=min\left({\left\{B_{i}\right\}}_{i=1,\ldots ,J}\right)$*bits*
2. Maximum number of bits changed:
  $B_{max}=max\left({\left\{B_{i}\right\}}_{i=1,\ldots ,J}\right)$*bits*
3. Mean number of bits changed:
  $\bar{B}=\frac{1}{J}\sum_{i=1}^{J}B_{i}$*bits*
4. Mean changed probability (mean of $HD_{i}\left(\%\right)$):
  $P=(\frac{\bar{B}}{u})\times 100$*%*
5. Standard variance of the changed bit number:
  $\Delta B=\sqrt{\frac{1}{J-1}\sum_{i=1}^{J}{\left(B_{i}-\bar{B}\right)}^{2}}$
6. Standard variance of the changed probability:
  $\Delta P=\sqrt{\frac{1}{J-1}\sum_{i=1}^{J}{\left(\frac{B_{i}}{u}-P\right)}^{2}}\times 100$*%*

The obtained results given in Table 11 with 2048 tests demonstrate that the diffusion effect is close to the expected results ($\bar{B}$ = 128 bits for the 256-bit hash value length, $\bar{B}$ = 256 for the 512-bit hash value length, and *P* = 50% for all proposed structures). In addition, it is noted that the diffusion is extremely stable for whatever the hash value length |h| in both Structure 1 and 2 because both the mean of number of changed bits $\bar{B}$ and the mean of changed probability *P* are very close to the ideal values, while ΔB and ΔP are very small.

For different number of tests (*J* = 512, 1024, and so on), similar results are obtained for the two proposed structures with their different hash value lengths (256 and 512 bits).

**Figure 12:**
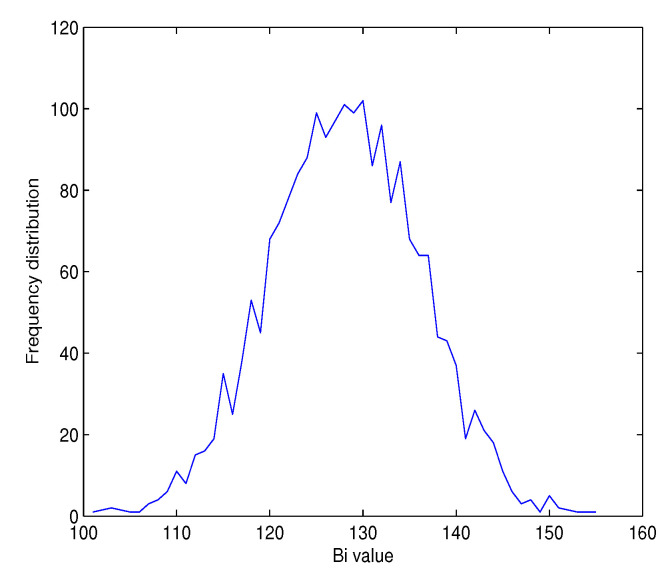
Histogram of $B_{i}$ for Structure 1 with |h| = 256 bits, and J = 2048 tests.

**Figure 13:**
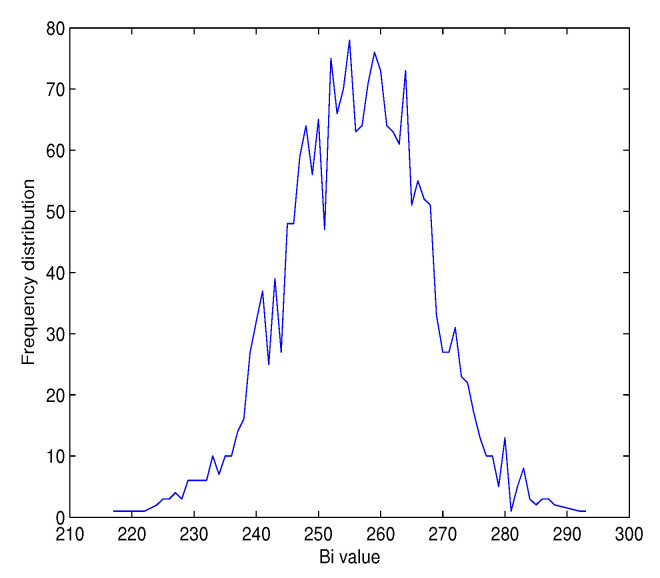
Histogram of $B_{i}$ for Structure 1 with |h| = 512 bits, and J = 2048 tests.

In addition, the histograms of $B_{i}$ as seen in Figure 12 and Figure 13 of Structure 1 illustrate that the values of $B_{i}$ are centered on the ideal values 128 and 256 bits for *u* = 256 and 512 bits, respectively. We obtain similar results for Structure 2.

### 4.3. Cryptanalysis

In the literature, there exist known attacks, which can be applied to the two categories of hash functions, unkeyed or keyed. In [24], *Bertoni* et al. demonstrate the dependency of these known attacks on the hash value length *u* for the unkeyed hash function with the secret key length |K| and for the keyed hash function with the hash value length *u*. Normally, if an attacker comprises the secret key *K*, then the system is completely compromised during the key life time [57]. In the following, the robustness of the proposed two structures, Structures 1 and 2, against these known attacks is demonstrated.

#### 4.3.1. Brute Force Attacks

The brute force attacks can be carried out on the secret key *K* (namely, *exhaustive key search attack*) and on the hash value *h*. We order the attacks on the hash value *h* from the easiest one to the hardest one:

1. Collision resistance attack
2. *Preimage attack* and *Second preimage attack*

*Exhaustive Key Search Attack* [58]:

With this kind of attack, the attacker needs $2^{|K|-1}$ = $2^{159}$ tries for the two proposed hash functions. Thus, this attack is ineffective.

*Collision Resistance Attack* (Birthday Attack) [59]:

With this kind of attack, the attacker tries to find two different messages $\left(M,M^{\prime }\right)$, which the proposed hash functions produce the same hash value *h*. To break the collision resistance property, the smaller workload expected by the attacker is approximately equal to $2^{u/2}$.

*Preimage and Second Preimage Attacks* [60]:

With the *Preimage* attack, the attacker tries to find the original message *M* for a known value *h* such that *H*(*M*) = *h*. In the *Second preimage* attack, knowing the hash value *h* for a given input message *M*, the attacker tries to find another message $M^{\prime }$ that produces the same hash value *h*. With these two types of attacks, the smaller expected workload required by the attacker to break the collision resistance property is approximately $2^{u}$.

In conclusion, to realize the attack on the hash value *h* for the two proposed structures with the minimum length (*u* = 256 bits), the minimum workload required by the attacker is $2^{128}$ attempts, which is infeasible.

#### 4.3.2. Cryptanalytic Attacks

With these kinds of attacks, the attacker tries to find specific weaknesses in the structure of a hash function, and performs on it some attacks, and it is expected that the amount of effort less than that with the brute force attack. In the next paragraphs, the two most common cryptanalytic attacks in the literature against the proposed hash functions are considered such that:

1. *Padding attack* (*Length extension attack*)
2. Meet-in-the-middle (MITM) preimage attack

*Padding Attack* [61]:

In the two proposed hash functions, the secret key *K* is used as an input for the *CS* to produce the necessary supplies to the *CNN*, and is not prepended to the message *M*. Then, this type of attack cannot be conducted.

*Meet-in-the-Middle Preimage Attack* [62]:

The Meet-in-the-middle (*MITM*) attack is a generic cryptanalytic approach that is originally applied to the cryptographic systems based on block ciphers (chosen-plaintext attack). In 2008, *Aoki* and *Sasaki* [62] noticed that the *MITM* attack could be applied to hash functions, to find collision, preimage, or second preimage for intermediate hash chaining values instead of the final hash value *h*. This attack has successfully broken several hash function designs. As our hash functions are preimage resistant, the minimum effort (with *u* = 256 bits) to succeed the *MITM* attack with probability 0.632 is $2^{u/2}=2^{128}$ tries.

### 4.4. Computing and Complexity Analysis

**Figure 14:**
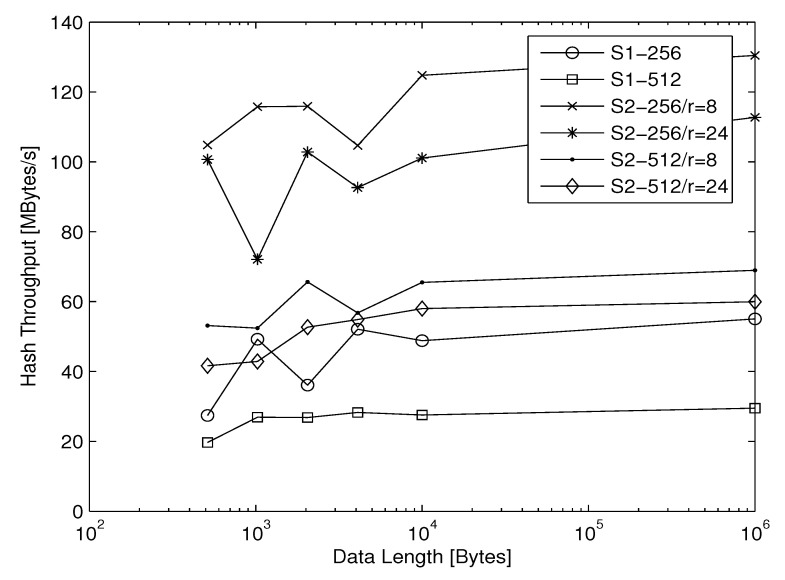
Comparison of hashing throughput for Structures 1 and 2 - $n_{r}$ = 8/24 rounds with |h| = 256/512 bits.

Here, the computing performance and the computational complexity of the two proposed structures are analyzed. Firstly, the computing performance of the two proposed structures with their hash value lengths of 256 and 512 bits for different message lengths is estimated. Then, the average hashing throughput *HTH* [MBytes/second] and the needed number of cycles to hash one Byte *NCpB* [cycles/byte] are calculated by Equations (27) and (28), respectively, as

(27) $$HTH\;\left[MBytes/s\right]=\frac{\left|M|[MBytes\right]}{HT[s]},$$

(28) $$NCpB\;\left[cycles/Byte\right]=\frac{CPUspeed[Hz]}{HTH[Byte/s]},$$

where *HT* [second] is the average hashing time. The calculation is done in C code, running an Ubuntu Linux 14.04.1 (64-bit) operating system and using a computer with a 2.9 GHZ Intel core i7-4910MQ CPU and with 4 GB of RAM. In Table 12 and Table 13, the average *HT*, the average *HTH*, and the average *NCpB* for the two proposed structures with their two hash value lengths of 256 and 512 bits are given. When the overhead related to the structures becomes negligible (from 10,000 data bytes and more), we observe that for any length of the hash values (256 or 512 bits), the hash throughput *HTH* of Structure 2 is just over twice that compared to Structure 1. In addition, we observe that, with any proposed structure, the hash throughput *HTH* with |h| equal to 256 bits (*r* = 1088 bits and *c* = 512 bits) is approximately twice the value with |h| equal to 512 bits (*r* = 576 bits and *c* = 1024 bits). Indeed, when *r* is increased, the hash time *HT* of the absorbing phase is decreased. Additionally, the *HTH* for the two proposed structures with their different hash value lengths are shown in Figure 14.

In addition, the computational complexity of the proposed functions varies with the number of required instructions and the latency of executions of these instructions. The computational complexity can be estimated by the big-*O* notation, which excludes constants, coefficients, and lower order terms. Indeed, the complexity is represented as a function *O*(*f*(*n*)) that depends on the input size *n*. It should be noted that the complexity of a series of sentences is in the same order of the sum of the individual complexities. In addition, some practical rules are considered to calculate the complexity [63] as

1. Input–output simple sentences are on the order of *O*(1).
2. *If* sentences are on the order of *O*(1).
3. *For* cycle is on the order of *O*(1) for *k* iterations independent of the input *n* or on the order of *O*(*n*) for *n* iterations.
4. *For* double nested cycle is on the order of *O*($n^{2}$) for *n* iterations for each cycle.
5. Iterative cycles with divisive-multiplicative sentences are on the order of *O*(*log n*) for *n* iterations.
6. *O*(*log n*) in the *For* cycle with *n* iterations is on the order of *O*(*n log n*).

The two proposed hash functions (Structures 1 and 2) are based on *Sponge* construction. These proposed hash functions are built as follows:

1. The hashing process starts by taking a block message with fixed length as input.
2. The message block is padded using a cryptographically secure padding scheme.
3. The padded message block is entered for a combination of operations with a key obtained from the output of the previous block.
4. The final hash block outputs a fixed length hash value having the same size as the input block.

In our proposed hash functions, the equations of the key generator, neural network layers, and nonlinear functions are realized by multiplication/division and addition/subtraction operations. In addition, *for* double nested operations are used. This means that the computational complexity of the two proposed hash functions is on the order of *O*($n^{2}$) [64,65].

### 4.5. Performance Comparison with the Standards SHA3, SHA2, and with Other Chaos-Based Hash Functions

This section presents the comparison of the computing performance for our proposed hash functions with the standard hash functions *SHA-3*, *SHA-2* and some chaos-based hash functions in the literature in terms of robustness and speed. To the best of our knowledge, there has not been any chaos-based hash function using *Sponge* construction in the literature.

In Table 14, Table 15, Table 16, Table 17 and Table 18, we compare the obtained statistical results (collision resistance, diffusion, and message sensitivity) of our proposed chaos-based hash functions with the standard *SHA-3* for |h| with the lengths of 256 and 512 bits, and with the standard *SHA-2* and some other chaos-based hash functions in the literature for |h| equal to 256 bits. We can conclude that, after carefully analyzing the values in these tables, all of our obtained statistical results are close to those of the standard *SHA-3* and of the other hash functions.

A comparison in terms of the needed number of cycles to hash one byte (*NCpB*) of the proposed chaos-based hash functions with the standard *SHA-3* for 2048 tests and different data sizes is given in Table 19. We observe that globally the performance of the standard hash algorithm *SHA-3* in terms of *NCpB* is better than that obtained by the proposed our hash functions. For example, for the long messages with length equal to 1 MB, the *NCpB* obtained by *SHA-3* for both the hash length value, is seven times less than the *NCpB* of Structure 1, but it is only less than three times of the *NCpB* obtained by Structure 2 with $n_{r}$ = 8. However, we do our simulations in the sequential implementation without optimization. Thus, with a parallel implementation (with 50 output neurons at the input layer) using optimized calculation, the performance computing will be at least similar to that obtained on *SHA-3* [66]. It can be even better than that of *SHA-3* when using our proposed Structure 2 with $n_{r}$ = 8.

Finally, we give a comparison of *NCpB* of the proposed structures with 256-bit and 512-bit hash values with some chaos-based hash functions and with the standards *SHA-2* and *SHA-3* for one Mbits data size in Table 20. We observe that the obtained *NCpB* is better than the *NCpB* of the other cited works, except for that obtained in our previous work [47]. It is because the the structure of the *Sponge* construction is more complex than that of the *Merkle–D$\overset{˚}{a}$mgard* construction.

**Table 14:** Comparison of collision resistance for the two proposed structures with |h| = 256 bits with the standards *SHA-3*, *SHA-2* and with some chaos-based hash functions.

Hash Function	Number of Hits ω				Absolute Difference *d*				
	0	1	2	3		Mean	Mean/Character	Minimum	Maximum
Structure 1	1806	229	13	0		2715.39	84.85	1695	3831
Structure 2 with $n_{r}$ = 8	1787	244	17	0		2584.51	80.76	1654	3759
Structure 2 with $n_{r}$ = 24	1824	213	11	0		2665.24	83.28	1642	3784
Abdoun et al. **${\mathrm{Structure}}_{\mathrm{MD}}$ 1** [47]	1931	114	3	0		1291.64	80.72	480	2038
Abdoun et al. **${\mathrm{Structure}}_{\mathrm{MD}}$**2**-$n_{r}$ = 8** [47]	1929	114	5	0		1426.23	89.13	730	2213
Abdoun et al. **${\mathrm{Structure}}_{\mathrm{MD}}$**2**-$n_{r}$ = 24** [47]	1942	106	0	0		1338.85	83.67	629	2071
SHA3-256 [13]	1818	211	19	0		2776.16	86.75	1686	3895
SHA2-256 [27]	1817	220	11	0		2707.10	84.59	1789	3819
Xiao et al. [51]	-	-	-	-		1506	94.12	696	2221
Xiao et al. [67]	1926	120	2	0		1227.8	76.73	605	1952
Deng et al. [68]	1940	104	4	0		1399.8	87.49	583	2206
Yang et al. [69]	-	-	-	-		-	93.25	-	-
Xiao et al. [70]	1915	132	1	0		1349.1	84.31	812	2034
Li et al. [71]	1901	146	1	0		1388.9	86.81	669	2228
Wang et al. [72]	1917	126	5	0		1323	82.70	663	2098
Huang [73]	1932	111	5	0		1251.2	78.2	650	1882
Li et al. [74]	1928	118	2	0		1432.1	89.51	687	2220
Li et al. [3]	1899	124	25	0		1367.6	85.47	514	2221
Li et al. [75]	1920	124	4	0		1319.5	82.46	603	2149
He et al. [4]	1926	118	4	0		1504	94	683	2312
Xiao et al. [76]	1924	120	4	0		1431.3	89.45	658	2156
Yu-Ling et al. [77]	1928	117	3	0		1598.6	99.91	796	2418
Xiao et al. [78]	1932	114	2	0		1401.1	87.56	573	2224
Li et al. [79]	1920	122	6	0		-	-	-	-
Li et al. [80]	1905	135	8	0		1335	83.41	577	2089
Ahmad et al. [81]	1923	121	4	0		1364.7	85.29	537	2399
Li et al. [82]	1957	82	9	0		1425	89.07	646	2096
Lin et al. [83]	1931	114	3	0		-	90.23	-	-

**Table 15:** Comparison of collision resistance for the two proposed structures with the standard *SHA-3* for |h| = 512 bits.

Hash Function	Number of Hits ω					Absolute Difference *d*				
	0	1	2	3	4		Mean	Mean/Character	Minimum	Maximum
Structure 1	1572	419	51	6	0		5414.34	169.19	3911	7062
Structure 2 with $n_{r}$ = 8	1607	371	67	3	0		5478.30	171.19	3874	6871
Structure 2 with $n_{r}$ = 24	1600	399	46	2	1		5233.34	163.54	3767	6606
SHA3-512 [13]	1593	418	35	2	0		5502.66	171.95	3933	7106

**Table 16:** Comparison of the statistical results of diffusion effect for the two proposed structures with |h| = 256 bits with the standards *SHA-3*, *SHA-2* and with some chaos-based hash functions.

Hash Function	$B_{\min}$	$B_{\max}$	$\bar{B}$	*P(%)*	ΔB	ΔP%
Structure 1	101	155	128.10	50.04	7.96	3.11
Structure 2 with $n_{r}$ = 8	99	156	127.70	49.88	8.22	3.21
Structure 2 with $n_{r}$ = 24	99	154	127.88	49.95	8.02	3.13
Abdoun et al. **${\mathrm{Structure}}_{\mathrm{MD}}$ 1** [47]	100	154	127.95	49.98	8.03	3.13
Abdoun et al. **${\mathrm{Structure}}_{\mathrm{MD}}$ 2-$n_{r}$ = 8** [47]	103	157	127.97	49.99	8.01	3.13
Abdoun et al. **${\mathrm{Structure}}_{\mathrm{MD}}$ 2-$n_{r}$ = 24** [47]	100	157	127.88	49.95	7.94	3.10
SHA3-256 [13]	101	153	128.05	50.02	8.01	3.13
SHA2-256 [27]	104	154	128.01	50.00	7.94	3.10
Xiao et al. [51]	-	-	63.85	49.88	5.78	4.52
Lian et al. [52]	-	-	63.85	49.88	5.79	4.52
Zhang et al. [53]	46	80	63.91	49.92	5.58	4.36
Wang et al. [84]	-	-	63.98	49.98	5.53	4.33
Xiao et al. [67]	-	-	64.01	50.01	5.72	4.47
Deng et al. [85]	-	-	63.91	49.92	5.58	4.36
Deng et al. [68]	-	-	63.84	49.88	5.88	4.59
Yang et al. [69]	-	-	64.14	50.11	5.55	4.33
Xiao et al. [70]	-	-	64.09	50.07	5.48	4.28
Amin et al. [86]	-	-	63.84	49.88	5.58	4.37
Li et al. [71]	45	81	63.88	49.90	5.37	4.20
Wang et al. [72]	-	-	63.90	49.93	5.64	4.41
Akhavan et al. [87]	42	83	63.91	49.92	5.69	4.45
Huang [73]	-	-	63.88	49.91	5.75	4.50
Li et al. [74]	-	-	63.80	49.84	5.75	4.49
Wang et al. [88]	44	82	64.15	50.11	5.76	4.50
Li et al. [3]	-	-	63.56	49.66	7.42	5.80
Li et al. [75]	-	-	63.97	49.98	5.84	4.56
He et al. [4]	45	83	64.03	50.02	5.60	4.40
Jiteurtragool et al. [89]	43	81	62.84	49.09	5.63	4.40
Teh et al. [10]	-	-	64.01	50.01	5.61	4.38
Chenaghlu et al. [90]	-	-	64.12	50.09	5.63	4.41
Akhavan et al. [91]	43	82	63.89	49.91	5.77	4.50
Nouri et al. [92]	-	-	64.08	50.06	5.72	4.72
Xiao et al. [76]	47	83	63.92	49.94	5.62	4.39
Yu-Ling et al. [77]	-	-	64.17	50.14	5.74	4.49
Xiao et al. [78]	-	-	64.18	50.14	5.59	4.36
Li et al. [79]	-	-	64.07	50.06	5.74	4.48
Li et al. [80]	-	-	63.89	49.91	5.64	4.41
Ren et al. [93]	-	-	63.92	49.94	5.78	4.52
Guo et al. [94]	-	-	63.40	49.53	7.13	6.35
Yu et al. [95]	45.6	81.8	63.98	49.98	5.73	4.47
Zhang et al. [96]	-	-	64.43	49.46	5.57	4.51
Jiteurtragool et al. [89]	101	153	126.75	49.51	7.98	3.12
Chenaghlu et al. [90]	101	168	128.08	50.03	8.12	3.21
Teh et al. [97]	-	-	64.00	50.00	5.44	4.25
Li et al. [82]	45	84	64.27	50.21	5.59	4.36
Ahmad et al. [81]	45	82	63.87	49.90	5.58	4.36
Lin et al. [83]	-	-	64.10	50.08	5.58	4.36

**Table 17:** Comparison of the statistical results of diffusion effect for the proposed structures with the standard *SHA-3* for |h| = 512 bits.

Hash Function	$B_{\min}$	$B_{\max}$	$\bar{B}$	*P (%)*	ΔB	ΔP%
Structure 1	217	293	256.20	50.04	11.20	2.18
Structure 2 with $n_{r}$= 8	214	291	255.90	49.98	11.37	2.22
Structure 2 with $n_{r}$ = 24	215	296	255.53	49.90	11.41	2.23
SHA3-512 [13]	221	288	255.82	49.96	11.08	2.16

**Table 18:** Comparison of average $B_{i}$ and $HD_{i}\left(\%\right)$ for the sensitivity of the hash value to the message of the two proposed structures with the standard *SHA-3* for |h| equal to 256 and 512 bits.

	Length of Hash Values	$B_{i}$	${HD}_{i}\%$
Structure 1	256	137.60	53.75
	512	266.00	51.95
Structure 2	256	128.00	50.00
$n_{r}$ = 8	512	204.40	39.92
Structure 2	256	134.60	52.57
$n_{r}$ = 24	512	254.20	49.64
SHA-3 [13]	256	124.00	48.43
	512	248.00	48.43

**Table 19:** Comparison of *NCpB* of the two proposed structures with the standard *SHA-3* for |h| equal to 256 and 512 bits.

Message Length	Structure 1		Structure 2 with $n_{r}$ = 8		Structure 2 with $n_{r}$ = 24		SHA-3	
	256	512	256	512	256	512	256	512
513	124.33	172.47	30.24	54.61	28.20	75.04	13.53	59.39
1024	60.68	103.42	24.45	57.64	51.78	78.30	32.12	48.83
2048	93.56	107.66	24.43	42.32	27.08	57.99	27.10	41.22
4096	53.28	98.48	33.38	55.19	35.27	54.32	15.92	13.82
$10^{4}$	63.51	101.87	22.44	42.49	30.71	47.82	13.28	13.43
$10^{6}$	50.30	93.67	21.21	40.12	24.56	46.16	6.92	12.95

**Table 20:** Comparison of *NCpB* of Structures 1 and 2 with 256-bit and 512-bit hash values length with the standards *SHA-3* and *SHA-2* and with some chaos-based hash functions and.

Hash Function	Structure 1		Structure 2 with $n_{r}$ = 8		Structure 2 with $n_{r}$ = 24		SHA-3		SHA-2	
	256	512	256	512	256	512	256	512	256	512
***NCpB***	50.30	93.67	21.21	40.12	24.56	46.16	6.92	12.95	11.87	13.72
**Hash function**	**${\mathrm{Structure}}_{\mathrm{MD}}$ 1** [47]		${\mathrm{Structure}}_{\mathrm{MD}}$ 2 [47]		Wang [84]	Akhavan [87]	Teh [10]			
			$n_{r}$ = 8	$n_{r}$ = 24						
***NCpB***	30.85		15.24	16.25	122.4	105.5	28.45			

## 5. Conclusions and Future Work

In this paper, we have designed and realized the two proposed keyed *CNN* hash functions, conducted analysis of the computing performance, and performed security. These two structures are based on the *Sponge* construction and have two hash output lengths, i.e., 256 and 512 bits. The results of analysis in terms of cryptanalytical attacks and statistical analyses are similar to those obtained by the standard hash algorithm *SHA-3*. For the computing performance term, the results of our two proposed structures are less than the standard hash algorithm *SHA-3* due to the sequential implementation. For a parallel implementation using 50 output neurons [66], the computing performance of Structure 2 with $n_{r}$ = 8 will be better than *SHA-3*. Then, the proposed *keyed-Sponge CNN* hash functions can be used in Digital Signature, Message Authentication, and Data Integrity applications.

Our future work will focus on the Extendable-Output Functions (*XOF*s), based on the keyed-*Sponge CNN* (*CNN-SHAKE*), where the proposed structures can produce hash outputs with variable length (as per user request). In addition, we will design and realize a new *CNN* structure based on the *Duplex* construction (*CNN-DUPLEX*) that will be useful for Authenticated Encryption with Associated Data (*AEAD*) applications.

## Figures and Tables

**Table 1:** Characteristics of the two proposed *keyed-Sponge* hash functions based on *CNN*.

Hash Function	Characteristics			
	Definition	*r* (bits),	*c* (bits)	|h| (bits)
Structure 1-256 (M)	*KSCNN*[512] (M ‖ 01, 256)	1088	512	256
Structure 2-256 (M)				
Structure 1-512 (M)	*KSCNN*[1024] (M ‖ 01, 512)	576	1024	512
Structure 2-512 (M)				

**Table 2:** Theoretical values of ω with respect to the number of tests *J* for |h| = 256 bits.

		Number of Hits ω				
		0	1	2	3	32
J	512	451.72	56.68	3.44	0.13	$4.42\times 10^{-75}$
	1024	903.45	113.37	6.89	0.27	$8.84\times 10^{-75}$
	2048	1806.91	226.74	13.78	0.54	$1.76\times 10^{-74}$

**Table 3:** Theoretical values of ω with respect to the number of tests *J* for |h| = 512 bits.

		Number of Hits ω					
		0	1	2	3	4	64
J	512	398.55	100.02	12.35	1.00	0.05	$7.14\times 10^{-57}$
	1024	797.10	200.05	24.71	2.00	0.11	$1.42\times 10^{-56}$
	2048	1594.20	400.11	49.42	4.00	0.23	$2.85\times 10^{-56}$

**Table 4:** Number of hits ω with respect to the number of rounds $n_{r}$ of Structure 2 for 2048 tests.

		ω					
		0	1	2	3	4	5
	**$n_{r}$**						
**|h|**							
256	1	1814	220	14	0	0	0
	2	1815	224	8	1	0	0
	4	1802	232	13	1	0	0
	8	1787	244	17	0	0	0
	16	1825	214	8	1	0	0
	24	1824	213	11	0	0	0
512	1	1598	396	52	1	1	0
	2	1552	439	52	5	0	0
	4	1594	401	44	6	3	0
	8	1607	371	67	3	0	0
	16	1602	395	47	4	0	0
	24	1600	359	46	2	1	0

**Table 5:** Number of hits ω regarding the proposed structures with the two lengths of hash values for 2048 tests.

	|h|	ω				
		0	1	2	3	4
Structure 1	256	1806	229	13	0	0
	512	1572	419	51	6	0
Structure 2	256	1787	244	17	0	0
$n_{r}$ = 8	512	1607	371	67	3	0
Structure 2	256	1824	213	11	0	0
$n_{r}$ = 24	512	1600	399	46	2	1

**Table 6:** Mean, mean/character, minimum, and maximum of the absolute difference *d* for the proposed structures with the two lengths of hash values and *J* = 2048 tests.

	|h|	Mean	Mean/Character	Minimum	Maximum
Structure 1	256	2715.39	84.85	1695	3831
	512	5414.34	169.19	3911	7062
Structure 2	256	2584.51	80.76	1654	3759
$n_{r}$ = 8	512	5478.30	171.19	3874	6871
Structure 2	256	2665.24	83.28	1642	3784
$n_{r}$ = 24	512	5233.34	163.54	3767	6606

**Table 7:** Sensitivity of *h* to *M* for the proposed structures with |h| = 256 bits.

	Message Variants	Hash Values in Hexadecimal Format	$B_{i}$	${HD}_{i}\%$
Structure 1	1	d53280d1f7a652977e7943472ea34a343746f09f6c8ea084f0b9d5009fecf467	–	–
	2	2081268dee082e8b2a9cbaaa8156fad0595d6fbd83aea9a92a5c649d9e53a82e	139.00	54.29
	3	9c0f5327df3f01a4311283caae6051a7780ca06d81d69dbfdfed57dec4a67db4	128.00	50.00
	4	c0a1b6e48295f620c2c42e1ed101023cbefecf6eca5d505d3355604fb8bb2db0	142.00	55.46
	5	e3edfd704f2befe9b54c6d000b1116316112b98cf0b6432f68ddf0ee6b829fcf	133.00	51.95
	6	29f9cf09e3d0764b53c4a67a5450fc828fc78e12af51de43b6b77f978292cdb3	146.00	57.03
	Average	–	137.60	53.75
Structure 2	1	d3a15d8621f3fec42dca5abf7077091f96275130fcef4e21a1521d81470245ae	–	–
$n_{r}$ = 8	2	346dd0bf7ac39dd0992e27b4fdef79e6aacda0d29733324ef3f26c1ca4d0b528	133	51.95
	3	2ae7c91d1e34279fcc90fdee067837028045a922c786c55c0d6e0fb08b539190	133.00	51.95
	4	82ed73ae08e2efe8498d795a2fe685a730a5c2fdaec6dd8cc8ad2171d7ee662b	116.00	45.31
	5	3bae189d094240cf7ca3a5ffcf9846f056d078b4ba10f76d092b146290632a26	137.00	53.51
	6	145759fe7d944ed8adaa126d7d0107cef75326f757812c56872a39f50d7818cc	121.00	47.26
	Average	–	128.00	50.00
Structure 2	1	f39457de07d62bea3fb35b5698ec008e004db03197b77a7e30e821a6a8499119	–	–
$n_{r}$ = 24	2	cb5dc81199de92b10ebf54d31185f37676ba5ca36d077d91723dda34150275e1	140	54.68
	3	9a0d013b3132a1db0ada8a5aa59ce1a49d38137760d7dc81cf91b77ff73545ac	140.00	54.68
	4	ef73910049a7a86ace7103c7d8f537fdfab9eab130c81f0d264c2b370400f67b	122.00	47.65
	5	2087a2da6dcf4187ad407532ce2207c14673ff0e56d512fa35b76009bde698c6	128.00	50.00
	6	006b3905b48157204b5a2c0922cdb1a869a297e3add562abc442ff0a8f2dd941	143.00	55.85
	Average	–	134.60	52.57

**Table 8:** A comparison of average $B_{i}$ and $HD_{i}\left(\%\right)$ for the sensitivity of *h* to *M*.

	Length of Hash Values	$B_{i}$	${HD}_{i}\%$
Structure 1	256	137.60	53.75
	512	266.00	51.95
Structure 2	256	128.00	50.00
$n_{r}$ = 8	512	204.40	39.92
Structure 2	256	134.60	52.57
$n_{r}$ = 24	512	254.20	49.64

**Table 9:** Sensitivity of *h* to *K* for the two proposed structures with |h| = 256 bits.

	Message Variants	Hash Values in Hexadecimal Format	$B_{i}$	${HD}_{i}\%$
Structure 1	1	d53280d1f7a652977e7943472ea34a343746f09f6c8ea084f0b9d5009fecf467	–	–
	2	a3614a0d3d7d77cffbde676045f5abf4add0f46ec9ed08e293e2a96118bbb364	124.00	48.43
	3	9cc68e614f3ce3161ece75dc8474d31f7a080fb30b7edf239334fd485cb5e8ca	131.00	51.17
	4	5a2502125bc452c8d7ac3c4f20de5ee4f422219839bbfabf1a22923b2a87cb96	130.00	50.78
	5	ac84f96d784967e643d750f9c15184ab4e6a93c408bf5eca22585f99eb98fa31	146.00	57.03
	Average	–	132.75	51.85
Structure 2	1	d3a15d8621f3fec42dca5abf7077091f96275130fcef4e21a1521d81470245ae	–	–
$n_{r}$ = 8	2	5e148302c03950dffe19911bd144c5713ed1c8750bee6c8324b338e9cb2635ed	121.00	47.26
	3	f5d2f5ae0db1c67d5a85f47994ea894db129241c07a361a4c9cc1c90ec0fb1c1	122.00	47.65
	4	18eae0eac4dcdedc01b8d55e231119e1d5286bb2fa08f107d8a13db82e984feb	124.00	48.43
	5	b56c8b1b210b34cb5a41948d7e1b16ba90614af2c1c4d64ee59e54790be40831	128.00	50.00
	Average	–	123.75	48.33
Structure 2	1	f39457de07d62bea3fb35b5698ec008e004db03197b77a7e30e821a6a8499119	–	–
$n_{r}$ = 24	2	d920e5ea9ae97a63fc75bb205733bc329464c5c67f868620d4c081321797f8c6	141	55.07
	3	dce025ba7f9fb1b72d2754eeeafb696740d691fd3129744bf6f549c25cd8b158	115.00	44.92
	4	c5e3e27affb359a4648039f8201e029213eb9345f730cf66b3aef40c805b65db	119.00	46.48
	5	182bb7760e4708c3464bbaed011154a9d903f06be1d73d9ea68dd3da7e9f7718	130.00	50.78
	Average	–	126.25	49.31

**Table 10:** A comparison of average $B_{i}$ and $HD_{i}\left(\%\right)$ for for the sensitivity of *h* to *K*.

	Length of Hash Values	$B_{i}$	${HD}_{i}\%$
Structure 1	256	132.75	51.85
	512	252.50	49.31
Structure 2	256	123.75	48.33
$n_{r}$ = 8	512	265.50	51.85
Structure 2	256	126.25	49.31
$n_{r}$ = 24	512	256.00	50.00

**Table 11:** Statistical analysis of diffusion effect results for Structures 1 and 2, with the two lengths of hash values, and *J* = 2048 tests.

		Length of Hash Values	
		256	512
Structure 1	$B_{min}$	101	217
	$B_{max}$	155	293
	$\bar{B}$	128.10	256.20
	*P*	50.04	50.04
	ΔB	7.96	11.20
	ΔP	3.11	2.18
Structure 2	$B_{min}$	99	214
$n_{r}$ = 8	$B_{max}$	156	291
	$\bar{B}$	127.70	255.90
	*P*	49.88	49.98
	ΔB	8.22	11.37
	ΔP	3.21	2.22
Structure 2	$B_{min}$	99	215
$n_{r}$ = 24	$B_{max}$	154	296
	$\bar{B}$	127.88	255.53
	*P*	49.95	49.90
	ΔB	8.02	11.41
	ΔP	3.13	2.23

**Table 12:** *HT*, *HTH*, and *NCpB* for Structures 1 and 2 with |h| = 256 bits and 2048 random tests.

Message	Structure 1				Structure 2 with $n_{r}$ = 8				Structure 2 with $n_{r}$ = 24		
Length	*HT*	*HTH*	*NCpB*		*HT*	*HTH*	*NCpB*		*HT*	*HTH*	*NCpB*
513	0.0058	27.41	124.33		0.0019	104.81	30.24		0.0029	100.65	28.20
1024	0.0102	49.25	60.68		0.0039	115.78	24.45		0.0039	72.10	51.78
2048	0.0190	36.90	93.56		0.0078	115.90	24.43		0.0087	102.86	27.08
4096	0.0336	52.08	53.28		0.0156	104.67	33.38		0.0175	92.64	35.27
$10^{4}$	0.0849	48.84	63.51		0.0371	124.75	22.44		0.0419	101.10	30.71
$10^{6}$	8.2666	55.05	50.30		3.5986	130.45	21.21		4.0537	112.70	24.56

**Table 13:** H*HT*, *HTH*, and *NCpB* for Structures 1 and 2 with |h| = 512 bits and 2048 random tests.

Message	Structure 1				Structure 2 with $n_{r}$ = 8				Structure 2 with $n_{r}$ = 24		
Length	*HT*	*HTH*	*NCpB*		*HT*	*HTH*	*NCpB*		*HT*	*HTH*	*NCpB*
513	0.0097	19.68	172.47		0.0043	53.16	54.61		0.0043	41.65	75.04
1024	0.0180	26.93	103.42		0.0073	52.42	57.64		0.0087	42.87	78.30
2048	0.0336	26.84	107.66		0.0141	65.65	42.32		0.0161	52.71	57.99
4096	0.0698	28.30	98.48		0.0278	56.87	55.19		0.0336	54.85	54.32
$10^{4}$	0.1621	27.57	101.87		0.0712	65.50	42.49		0.0761	58.02	47.82
$10^{6}$	15.6166	29.53	93.67		6.6293	68.97	40.12		7.8032	59.95	46.16

## References

[1] Datcu O., Macovei C., Hobincu R. (2020). Chaos Based Cryptographic Pseudo-Random Number Generator Template with Dynamic State Change. Appl. Sci..

[2] Abdoun N. (2019). Design, Implementation and Analysis of Keyed Hash Functions Based on Chaotic Maps and Neural Networks. Ph.D. Thesis.

[3] Li Y., Xiao D., Deng S., Han Q., Zhou G. (2011). Parallel Hash function construction based on chaotic maps with changeable parameters. Neural Comput. Appl..

[4] He B., Lei P., Pu Q., Liu Z. A method for designing hash function based on chaotic neural network. Proceedings of the International Workshop on Cloud Computing and Information Security (CCIS).

[5] Levy D. (1994). Chaos theory and strategy: Theory, application, and managerial implications. Strateg. Manag. J..

[6] Rosenblatt F. (1958). The perceptron: A probabilistic model for information storage and organization in the brain. Psychol. Rev..

[7] Lorenz E.N. (1963). Deterministic nonperiodic flow. J. Atmos. Sci..

[8] Hilborn R.C. (2001). Chaos and Nonlinear Dynamics: An Introduction for Scientists and Engineers.

[9] Hoang T.M., Assad S.E. (2020). Novel Models of Image Permutation and Diffusion Based on Perturbed Digital Chaos. Entropy.

[10] Teh J.S., Samsudin A., Akhavan A. (2015). Parallel chaotic hash function based on the shuffle-exchange network. Nonlinear Dyn..

[11] National Institute of Standards and Technology, PUB FIPS (2012). 180-4. Secure Hash Standard. Federal Information Processing Standards Publication 180-4.

[12] Stevens M.M.J. (2012). Attacks on Hash Functions and Applications. Ph.D. Thesis.

[13] Dworkin M.J. (2015). SHA-3. Standard: Permutation-Based Hash and Extendable-Output Functions.

[14] Bertoni G., Daemen J., Peeters M., Van Assche G. (2011). Cryptographic Sponge Functions. Submiss. NIST (Round 3).

[15] Gauravaram P., Knudsen L.R., Matusiewicz K., Mendel F., Rechberger C., Schläffer M., Thomsen S.S. Grøstl-a SHA-3 candidate. Proceedings of the Dagstuhl Seminar Proceedings.

[16] Wu H. (2011). The Hash Function JH. Submiss. NIST (Round 3).

[17] Ferguson N., Lucks S., Schneier B., Whiting D., Bellare M., Kohno T., Callas J., Walker J. (2010). The Skein hash function family. Submiss. NIST (Round 3).

[18] Aumasson J.P., Meier W., Phan R.C.W., Henzen L. (2014). The Hash Function BLAKE.

[19] Lucks S. (2004). Design Principles for Iterated Hash Functions. IACR Cryptol. EPrint Arch..

[20] Merkle R.C., Charles R. (1979). Secrecy, Authentication, and Public Key Systems.

[21] Damgård I.B. (1989). A design principle for hash functions. Lecture Notes in Computer Science, Proceedings of the Conference on the Theory and Application of Cryptology.

[22] Dunkelman O., Biham E. A framework for iterative hash functions: Haifa. Proceedings of the 2nd NIST Cryptographich Hash Workshop, University of California.

[23] Nandi M., Paul S. (2010). Speeding up the wide-pipe: Secure and fast hashing. Lecture Notes in Computer Science, Proceedings of the Indocrypt, Hyderabad, India, 12–15 December 2010.

[24] Bertoni G., Daemen J., Peeters M., Van Assche G. Sponge functions. Proceedings of the ECRYPT Hash Workshop.

[25] Rivest R. (2020). The MD5 Message-Digest Algorithm; Retrieved August 31. RFC 1321.

[26] FIPS PUB (1995). Secure hash standard. Public Law.

[27] Standard S.H., FIPS P. (2002). 180-2. August.

[28] Abdoun N., El Assad S., Hammoud K., Assaf R., Khalil M., Deforges O. New keyed chaotic neural network hash function based on sponge construction. Proceedings of the 2017 12th International Conference for Internet Technology and Secured Transactions (ICITST).

[29] Duval S., Leurent G. (2019). Lightweight MACs from Universal Hash Functions. Lecture Notes in Computer Science, Proceedings of the International Conference on Smart Card Research and Advanced Applications, Rague, Czech Republic, 11–13 November 2019.

[30] Luykx A., Preneel B., Tischhauser E., Yasuda K. (2016). A MAC Mode for Lightweight Block Ciphers. Lecture Notes in Computer Science, Proceedings of the Fast Software Encryption, Bochum, Germany, 20–23 March 2016.

[31] Gong Z., Hartel P., Nikova S., Tang S.H., Zhu B. (2014). TuLP: A Family of Lightweight Message Authentication Codes for Body Sensor Networks. J. Comput. Sci. Technol..

[32] Jean-Philippe A., Bernstein D. (2012). SipHash: A fast short-input PRF. Lecture Notes in Computer Science, Proceedings of the Progress in Cryptology-INDOCRYPT, Kolkata, India, 9–12 December 2012.

[33] Aumasson J.P., Henzen L., Meier W., Naya-Plasencia M. (2010). Quark: A lightweight hash. Lecture Notes in Computer Science, Proceedings of the International Workshop on Cryptographic Hardware and Embedded Systems CHES, Santa Barbara, CA, USA, 17–20 August 2010.

[34] Guo J., Peyrin T., Poschmann A. (2011). The PHOTON family of lightweight hash functions. Lecture Notes in Computer Science, Proceedings of the Advances in Cryptology–CRYPTO 2011, Santa Barbara, CA, USA, 14–18 August 2011.

[35] Bogdanov A., Knežević M., Leander G., Toz D., Varıcı K., Verbauwhede I. (2011). SPONGENT: A Lightweight Hash Function. Lecture Notes in Computer Science, Proceedings of the Cryptographic Hardware and Embedded Systems–CHES 2011, Nara, Japan, 28 September–1 October 2011.

[36] El Assad S., Noura H. (2014). Generator of Chaotic Sequences and Corresponding Generating System. U.S. Patent.

[37] Bashir I., Ahmed F., Ahmad J., Boulila W., Alharbi N. (2019). A Secure and Robust Image Hashing Scheme Using Gaussian Pyramids. Entropy.

[38] Bertoni G., Daemen J., Peeters M., Van Assche G. On the Security of the Keyed Sponge Construction. Proceedings of the Symmetric Key Encryption Workshop.

[39] Chang D., Dworkin M., Hong S., Kelsey J., Nandi M. A Keyed Sponge Construction with Pseudorandomness in the Standard Model. Proceedings of the Third SHA-3 Candidate Conference.

[40] Mennink B., Reyhanitabar R., Vizár D. (2015). Security of Full-state Keyed Sponge and Duplex: Applications to Authenticated Encryption. Lecture Notes in Computer Science, Proceedings of the International Conference on the Theory and Application of Cryptology and Information Security, Auckland, New Zealand, 29 November–3 December 2015.

[41] Andreeva E., Daemen J., Mennink B., Van Assche G. (2015). Security of keyed sponge constructions using a modular proof approach. Lecture Notes in Computer Science, Proceedings of the International Workshop on Fast Software Encryption, Istanbul, Turkey, 8–11 March 2015.

[42] Naito Y., Yasuda K. (2016). New Bounds for Keyed Sponges with Extendable Output: Independence between Capacity and Message Length. Lecture Notes in Computer Science, Proceedings of the International Conference on Fast Software Encryption, Bochum, Germany, 20–23 March 2016.

[43] Bertoni G., Daemen J., Peeters M., Van Assche G. Permutation-based encryption, authentication and authenticated encryption. Proceedings of the Directions in Authenticated Ciphers, (DIAC 2012).

[44] Gaži P., Pietrzak K., Tessaro S. (2015). The exact PRF security of truncation: Tight bounds for keyed sponges and truncated CBC. Lecture Notes in Computer Science, Proceedings of the Annual Cryptology Conference, Santa Barbara, CA, USA, 16–20 August 2015.

[45] Daemen J., Mennink B., Van Assche G. (2017). Full-state keyed duplex with built-in multi-user support. Lecture Notes in Computer Science, Proceedings of the International Conference on the Theory and Application of Cryptology and Information Security, Hong Kong, China, 3–7 December 2017.

[46] Mennink B. (2018). Key Prediction Security of Keyed Sponges. IACR Trans. Symmetric Cryptol..

[47] Abdoun N., El Assad S., Deforges O., Assaf R., Khalil M. (2020). Design and security analysis of two robust keyed hash functions based on chaotic neural networks. J. Ambient Intell. Humaniz. Comput..

[48] El Assad S. Chaos based information hiding and security. Proceedings of the 2012 International Conference for Internet Technology And Secured Transactions.

[49] Lee S.H., Kwon K.R., Hwang W.J., Chandrasekar V. (2013). Key-dependent 3D model hashing for authentication using heat kernel signature. Digit. Signal Process..

[50] Bellare M., Namprempre C. (2000). Authenticated encryption: Relations among notions and analysis of the generic composition paradigm. Lecture Notes in Computer Science, Proceedings of the International Conference on the Theory and Application of Cryptology and Information Security, Kyoto, Japan, 3–7 December 2000.

[51] Xiao D., Liao X., Deng S. (2005). One-way Hash function construction based on the chaotic map with changeable-parameter. Chaos Solitons Fractals.

[52] Lian S., Sun J., Wang Z. (2006). Secure hash function based on neural network. Neurocomputing.

[53] Zhang J., Wang X., Zhang W. (2007). Chaotic keyed hash function based on feedforward–feedback nonlinear digital filter. Phys. Lett. A.

[54] Preneel B. (1993). Analysis and Design of Cryptographic Hash Functions. Ph.D. Thesis.

[55] Shannon C.E. (1949). Communication theory of secrecy systems. Bell Syst. Tech. J..

[56] Feistel H. (1973). Cryptography and computer privacy. Scienfitic Am..

[57] Mironov I. (2005). Hash Functions: Theory, Attacks, and Applications.

[58] Bakhtiari S., Safavi-Naini R., Pieprzyk J. (1995). Cryptographic Hash Functions: A Survey.

[59] Flajolet P., Gardy D., Thimonier L. (1992). Birthday paradox, coupon collectors, caching algorithms and self-organizing search. Discret. Appl. Math..

[60] Chen S., Jin C. (2018). Preimage Attacks on Some Hashing Modes Instantiating Reduced-Round LBlock. IEEE Access.

[61] Hash Length Extension Attacks|Java Code Geeks-2017. https://www.javacodegeeks.com/2012/07/hash-length-extension-attacks.html.

[62] Aoki K., Sasaki Y. (2009). Meet-in-the-middle preimage attacks against reduced SHA-0 and SHA-1. Advances in Cryptology-CRYPTO 2009.

[63] Seok B., Park J., Park J.H. (2019). A lightweight hash-based blockchain architecture for industrial IoT. Appl. Sci..

[64] Arora S., Barak B. (2009). Computational Complexity: A Modern Approach.

[65] Mansour Y., Nisan N., Tiwari P. (1993). The computational complexity of universal hashing. Theor. Comput. Sci..

[66] Przytula K.W., Prasanna V.K., Lin W.M. (1992). Parallel implementation of neural networks. J. VLSI Signal Process. Syst. Signal Image Video Technol..

[67] Xiao D., Liao X., Wang Y. (2009). Parallel keyed hash function construction based on chaotic neural network. Neurocomputing.

[68] Deng S., Li Y., Xiao D. (2010). Analysis and improvement of a chaos-based Hash function construction. Commun. Nonlinear Sci. Numer. Simul..

[69] Yang H., Wong K.W., Liao X., Wang Y., Yang D. (2009). One-way hash function construction based on chaotic map network. Chaos Solitons Fractals.

[70] Xiao D., Liao X., Wang Y. (2009). Improving the security of a parallel keyed hash function based on chaotic maps. Phys. Lett. A.

[71] Li Y., Xiao D., Deng S. (2012). Secure hash function based on chaotic tent map with changeable parameter. High Technol. Lett.

[72] Wang Y., Du M., Yang D., Yang H. One-Way Hash Function Construction Based on Iterating a Chaotic Map. Proceedings of the International Conference on Computational Intelligence and Security Workshops 2007.

[73] Huang Z. (2011). A more secure parallel keyed hash function based on chaotic neural network. Commun. Nonlinear Sci. Numer. Simul..

[74] Li Y., Deng S., Xiao D. (2011). A novel Hash algorithm construction based on chaotic neural network. Neural Comput. Appl..

[75] Li Y., Xiao D., Deng S., Zhou G. (2013). Improvement and performance analysis of a novel hash function based on chaotic neural network. Neural Comput. Appl..

[76] Xiao D., Liao X., Deng S. (2008). Parallel keyed hash function construction based on chaotic maps. Phys. Lett. A.

[77] Yu-Ling L., Ming-Hui D. (2012). One-way hash function construction based on the spatiotemporal chaotic system. Chin. Phys. B.

[78] Xiao D., Shih F.Y., Liao X. (2010). A chaos-based hash function with both modification detection and localization capabilities. Commun. Nonlinear Sci. Numer. Simul..

[79] Li Y., Xiao D., Li H., Deng S. (2012). Parallel chaotic Hash function construction based on cellular neural network. Neural Comput. Appl..

[80] Li Y., Xiao D., Deng S. (2012). Keyed hash function based on a dynamic lookup table of functions. Inf. Sci..

[81] Ahmad M., Khurana S., Singh S., AlSharari H.D. (2017). A simple secure hash function scheme using multiple chaotic maps. 3D Res..

[82] Li Y., Li X., Liu X. (2017). A fast and efficient hash function based on generalized chaotic mapping with variable parameters. Neural Comput. Appl..

[83] Lin Z., Guyeux C., Yu S., Wang Q., Cai S. (2017). On the use of chaotic iterations to design keyed hash function. Clust. Comput..

[84] Wang Y., Liao X., Xiao D., Wong K.W. (2008). One-way hash function construction based on 2D coupled map lattices. Inf. Sci..

[85] Deng S., Xiao D., Li Y., Peng W. (2009). A novel combined cryptographic and hash algorithm based on chaotic control character. Commun. Nonlinear Sci. Numer. Simul..

[86] Amin M., Faragallah O.S., El-Latif A.A.A. (2009). Chaos-based hash function (CBHF) for cryptographic applications. Chaos Solitons Fractals.

[87] Akhavan A., Samsudin A., Akhshani A. (2009). Hash function based on piecewise nonlinear chaotic map. Chaos Solitons Fractals.

[88] Wang Y., Wong K.W., Xiao D. (2011). Parallel hash function construction based on coupled map lattices. Commun. Nonlinear Sci. Numer. Simul..

[89] Jiteurtragool N., Ketthong P., Wannaboon C., San-Um W. A Topologically Simple Keyed Hash Function Based on Circular Chaotic Sinusoidal Map Network. Proceedings of the 2013 15th International Conference on Advanced Communications Technology (ICACT).

[90] Chenaghlu M.A., Jamali S., Khasmakhi N.N. (2016). A novel keyed parallel hashing scheme based on a new chaotic system. Chaos Solitons Fractals.

[91] Akhavan A., Samsudin A., Akhshani A. (2013). A novel parallel hash function based on 3D chaotic map. EURASIP J. Adv. Signal Process..

[92] Nouri M., Khezeli A., Ramezani A., Ebrahimi A. A dynamic chaotic hash function based upon circle chord methods. Proceedings of the 6th International Symposium on Telecommunications (IST).

[93] Ren H., Wang Y., Xie Q., Yang H. (2009). A novel method for one-way hash function construction based on spatiotemporal chaos. Chaos Solitons Fractals.

[94] Guo X.F., Zhang J.S. (2006). Keyed one-way Hash function construction based on the chaotic dynamic S-Box. Acta Phys. Sin..

[95] Yu H., Lu Y.F., Yang X., Zhu Z.L. One-Way Hash Function Construction Based on Chaotic Coupled Map Network. Proceedings of the 2011 Fourth International Workshop on Chaos-Fractals Theories and Applications.

[96] Zhang H., Wang X.F., Li Z.H., Liu D.H. (2005). One way hash function construction based on spatiotemporal chaos. Acta Phys. Sin..

[97] Teh J.S., Tan K., Alawida M. (2019). A chaos-based keyed hash function based on fixed point representation. Clust. Comput..

